# Deep‐UV Light‐Emitting Based on the hBN:S/hBN: Mg Homojunction

**DOI:** 10.1002/advs.202414353

**Published:** 2025-03-16

**Authors:** Ransheng Chen, Qiang Li, Wannian Fang, Qifan Zhang, Jiaxing Li, Zhihao Zhang, Kangkang Liu, Feng Yun, Yanan Guo, Tao Wang, Yue Hao

**Affiliations:** ^1^ Key Laboratory of Physical Electronics and Devices for Ministry of Education and Shaanxi Provincial Key Laboratory of Photonics & Information Technology Xi'an Jiaotong University Xi'an 710049 China; ^2^ State Key Laboratory of Solid‐State Lighting Institute of Semiconductors and Key Laboratory of Semiconductor Materials Science Institute of Semiconductors Chinese Academy of Sciences Beijing 100083 China; ^3^ School of Physics and Astronomy Cardiff University Cardiff CF24 3AA UK; ^4^ School of Microelectronics Xidian University Xi'an 710126 China

**Keywords:** DFT calculations, hexagonal boron nitride, homojunction, sulfur doping

## Abstract

A hexagonal boron nitride (hBN) based p‐n homo‐junction is expected to demonstrate a great potential for being fabricated into an emitter (either light‐emitting diode or laser diode) in the deep‐UV spectral region. However, it remains a great challenge to achieve n‐type conductive hBN. Herein, n‐type hBN is obtained by means of doping sulfur into hBN. The structure and the electric properties of S‐doped hBN is studied via density functional theory, indicating that the orbital coupling between S 3p and B 2p orbital introduces shallow donor energy levels. The S atoms in the multilayer structure demonstrate enhanced electron delocalization compared with its mono‐layer counterpart, suggesting that multilayer hBN:S is more inclined to be n‐type conductive than its mono‐layer counterpart. Experimentally, a multilayer hBN:S sample is successfully grown on sapphire substrates, where the S content, up to 1.21%, is obtained. The hBN:S film shows an in‐plane current of 1.6 nA using Ti as ohmic contact and 8.4 nA using Ni as Schottky contact, respectively. The donor level induced by the S atoms is located at 0.349 eV below the CBM. Finally, a vertically‐stacked n‐hBN/p‐hBN (hBN:S/hBN: Mg) structured junction is grown, and demonstrating a promise for being fabricated into a deep‐UV emitter.

## Graphical abstract

S‐atoms are selected as donor dopants to achieve the n‐type conductivity of the hBN film. A theoretical model of S‐doping hBN using DFT calculation and experimentally prepared S‐doped hBN multilayers with a recorded doping concentration of 1.21% is established. Combining with Mg‐doped hBN films, a vertically‐stacked n‐hBN/p‐hBN homojunction, which shows the superiority of deep‐UV light‐emitting is constructed.

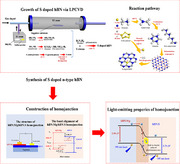

## 1 Introduction

As an emerging group‐III nitride‐based ultra‐wide band gap semiconductor, hexagonal boron nitride (hBN) has a bandgap of ≈5.9 eV.^[^ ^1^, ^2^, ^3^ ^]^ The hBN shows a 2D layered structure^[^ ^4^ ^]^ high band‐edge absorption coefficient,^[^ ^5^ ^]^ deep‐UV emission,^[^ ^6^, ^7^ ^]^ deep‐UV birefringent,^[^ ^8^ ^]^ and high electric breakdown strength.^[^ ^9^ ^]^ These properties promote hBN as a promising candidate for being fabricated into high‐power field‐effect devices, deep‐UV (DUV) optoelectronics, and solar‐blind communication^[^ ^10^, ^11^, ^12^, ^13^, ^14^ ^]^ In contrast to MoS_2_/SiO_2_, the field‐effect transistor consists of the MoS_2_/hBN heterostructure performs a notable advantages in carrier mobility and electron concentration.^[^ ^13^, ^15^ ^]^ The hBN‐based heterostructure is also expected to play an important role in the high sensitivity of vacuum UV photodetection^[^ ^16^, ^17^ ^]^ and neutron detection applications.^[^ ^5^, ^18^ ^]^ Furthermore, heterostructures, such as AlN/hBN,^[^ ^19^ ^]^ GaN/hBN^[^ ^20^ ^]^ and AlGaN/hBN,^[^ ^21^ ^]^ also display advantages in the fabrication of emitters in the UV spectral region, as hBN shows zero polarization fields. Recent studies showed that defect‐assisted DUV luminescence can be generated from hBN mainly due to stacking faults and point defects,^[^ ^6^, ^7^, ^22^ ^]^ demonstrating the great potential of hBN on light‐emitting materials. Generally, hBN materials for optical application are chemically synthesized hBN nanosheets or mechanically exfoliated hBN flakes from pyrolytic bulk materials. The hBN material is small in area and random in shape, which is difficult to meet the requirements of large‐scale device preparation, and is incompatible with traditional preparation techniques. Additionally, hBN can be fabricated as an emitter with high efficiency in the DUV region once a p‐n junction structure is achieved. However, a number of fundamental challenges, such as process incompatibility, lattice mismatch, and severe nonradiative recombination at the interface of a heterojunction, pose a huge limitation to the formation of heterostructure‐based photonics. As an initial step, it is necessary to achieve double type conductive of hBN at least so that a homojunction LED can be formed.

An undoped hBN film usually shows p‐type conductivity due to the existence of boron (B) vacancies.^[^ ^19^, ^23^, ^24^ ^]^ From the point of view of the negative electron affinity,^[^ ^25^, ^26^, ^27^, ^28^ ^]^ it is expected that hBN is more prone for accepting acceptors leading to p‐type conductivity than AlN or its alloys. For instance, Mg‐atoms incorporation has been demonstrated to be an effective acceptor dopant in hBN, leading to p‐type conductivity.^[^ ^21^, ^29^, ^30^, ^31^ ^]^ Carbon atoms which typically stem from metalorganic precursors during material deposition can substitute N atoms in hBN, which produces a deep acceptor energy level (2.3 eV).^[^ ^32^, ^33^ ^]^ Moreover, more comprehensive studies have discovered that IV‐group atoms (either carbon or silicon) doped into hBN tend to occupy both the B site and N site, thus forming the donor and the acceptor energy levels simultaneously,^[^ ^32^, ^34^, ^35^ ^]^ where the donor level can be largely offset by the acceptor impurities via compensation effects thus leading to p‐type conductivity in C or Si‐doped hBN.^[^ ^33^ ^]^ Although a pretty talent of hBN in acceptor doping, there is still a hurdle toward donor doping due to its high CBM.

IV‐group atoms can serve as either a donor or an acceptor, whereas VI‐ and VII‐group atoms tend to act as donors in hBN. Specifically, oxygen (O), fluorine (F), sulfur (S), and chlorine (Cl) with a relatively small atom radius are regarded as possible candidates to substitute the N atom of hBN. By assessing the electronegativity of a number of atoms (O = 3.44, F = 3.98, S = 2.58, Cl = 3.16), S atoms can act as donors with a low ionization energy in hBN. However, the commonly used metal foils (Cu,^[^ ^36^ ^]^ Fe,^[^ ^37^ ^]^ Ni,^[^ ^38^ ^]^ etc.) for hBN epitaxy inevitably react with S atoms to form metal sulfide, making it difficult for S‐atoms doping into the host lattice. The substitutional defects in hBN usually have a high formation energy which is higher than 2.5 eV,^[^ ^23^, ^39^ ^]^ thus requesting a high temperature for doping processes. This poses a great restriction because it is well‐known that most metal substrates cannot stand at a high temperature above 1200 °C.

In this study, a multilayer S‐doped hBN (hBN:S) sample was prepared on a sapphire substrate using a low‐pressure chemical vapor deposition (LPCVD) technique, where the redundant reaction between the metal substrate and the S precursor can be eliminated. The large‐area high‐crystallinity of hBN:S film is compatible with chip preparation techniques, thus enabling high repeatability preparation. Taking advantage of the high melting point of the sapphire substrate, doping processes at high temperatures, such as 1300 °C or above, can be possibly conducted. The electrical property of multilayer hBN:S was investigated by using DFT, confirming that S‐atoms can be used as efficient donors in hBN. The enhanced electron delocalization of the S atom has been observed in a multilayer structure than that in a mono‐layer hBN: S. Both the theoretical study and the experimental data demonstrated a variation in band gap and interlayer space in multilayer hBN film after S doping. Experimentally, by optimizing the growth temperature, a multilayer hBN:S film on sapphire displays an efficient S‐doping with a concentration of 1.21%. The S doping can suppress B_i_ and V_N_ defects effectively, therefore improving the crystallinity of the film. In contrast to the insulation characteristic of hBN, the hBN:S film shows a n‐type conductivity and a significantly improved surface current. The donor energy level induced by S‐atoms had been ascertained to have an ionization energy (*E*_D_) of 0.349 eV. Finally, a vertically‐stacked n‐hBN/p‐hBN (hBN:S/hBN: Mg) homojunction has been achieved, showing a light‐emitting property in the deep‐UV spectral region. The results obtained in this study pave a new way for the design and fabrication of hBN‐based optoelectronics in the UV spectral region.

## 2 Results and Discussion

**Figure 1:**
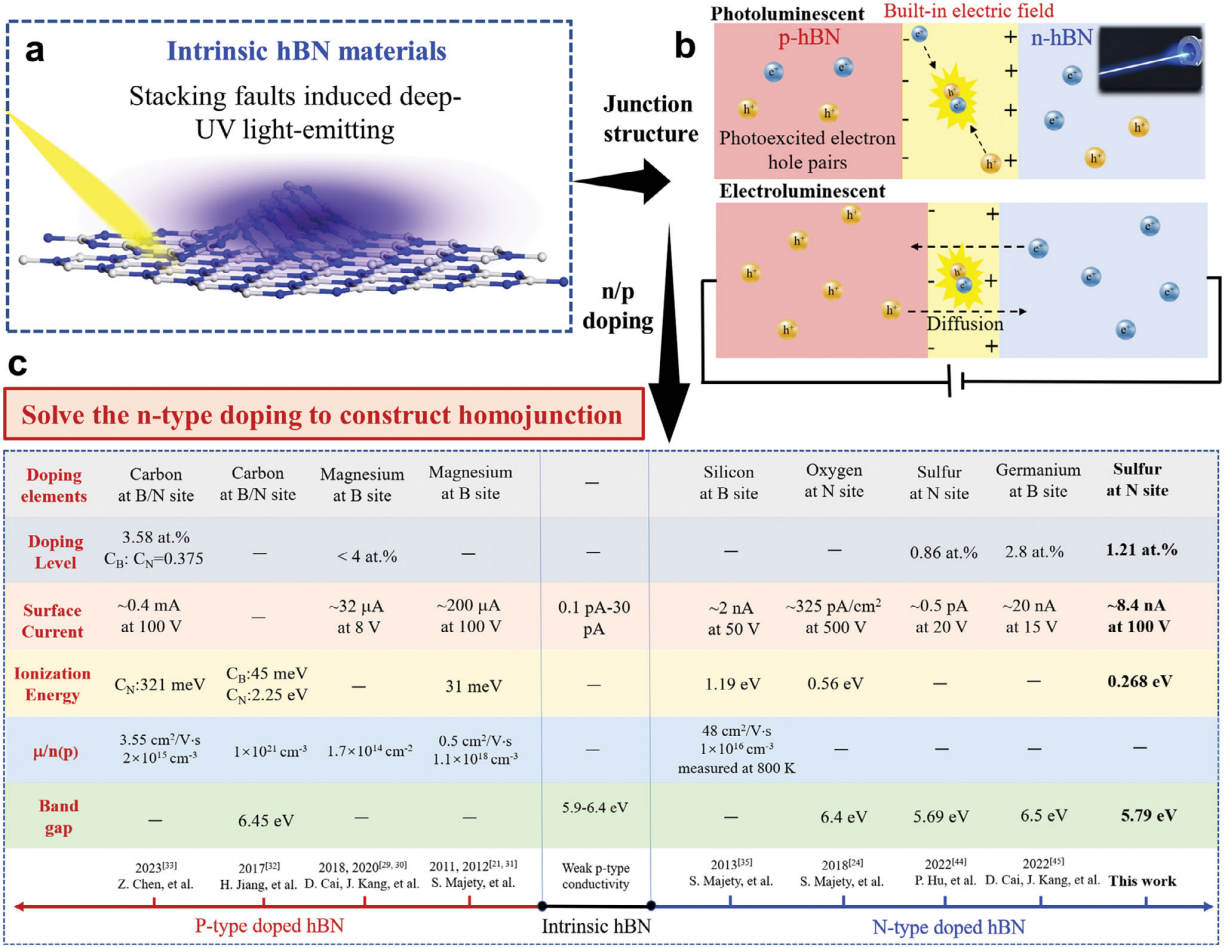
DUV optoelectronic process and doping characteristic of hBN. a) The luminescence model of the hBN crystal assisted by the faceted fold. b) Schematics of the photoluminescence and electroluminescence processes in hBN‐based homojunction. c) Doping characteristic comparison of hBN materials system, including n‐type and p‐type doping.

Because of the 2D layered nature of hBN, a high density of stacking faults is expected in a multiplayer structure due to its low activation energy. Our previous work has found that the multilayer hBN grown on sapphire, the under‐layer hBN was wrinkle‐free and stress‐free, while the upper‐layer hBN showed a significant number of wrinkles.^[^ ^40^, ^41^ ^]^ As shown in **Figure** **1**a, a more notable change in atom stacking order and stacking faults within hBN can be induced due to the generation of wrinkles under the folding processes. It also has been found that the defects‐assisted luminescence shows much stronger intensity than the intrinsic luminescence, and the most prominent luminescence comes from the layered stacking fault.^[^ ^6^, ^7^, ^42^, ^43^ ^]^ Defects‐assisted luminescence of hBN is within the DUV regime, and different defect types and stacking configurations can lead to different emissions wavelengths.^[^ ^22^ ^]^ Therefore, hBN can be used for the fabrication of DUV emitters. However, luminescence properties of hBN were evidenced in previous works by using monocrystal hBN nano flakes with a size of tens of micrometers that contradicts with ensuring device design. Large‐area hBN film via CVD epitaxy technique is more likely compatible with large‐scale device preparation, however, the luminescence characteristics of hBN film must have a notable discrepancy between monocrystal hBN nanosheet due to the different film characteristics (crystallinity, morphology, stacking fault stypes, etc.). The light‐emitting property of large‐scale hBN film prepared on sapphire deserves to study.

The construction of light‐emitting diodes (LED) is the foundation for the development of hBN‐based DUV light‐emitting device applications. Unlike hBN materials that will emit light assisted by near band edge free‐exciton recombination and bound‐exciton within defects under light injection or electrical injection, the diode structure has a different luminescence pattern assisted by space charge regions as shown in Figure 1b. The photoluminescence of p‐n homojunction is the photo‐excited electron–hole pairs that drift into the space charge region driven by the built‐in electric field, resulting in radiative recombination luminescence. The electroluminescence occurs when the intensity of the built‐in electric field is suppressed under the forward bias, and then the majority carrier continues to diffuse to the other side coupled with a radiative recombination luminescence.

Both p‐doping and n‐doping are requested to form a p‐n junction, and Figure 1c shows the typical doping results. Intrinsic hBN shows a weak p‐type conductivity, and the carbon element holds the ability to achieve p‐type doping but simultaneously substitutes B and N sites. Numerous studies demonstrated Mg in hBN is a predominant p‐type dopant, while it still remains a challenge to achieve n‐type doping, which is the primary limitation for the preparation of a hBN‐based p‐n junction.^[^ ^44^, ^45^ ^]^ In this work, a sulfur atom is selected as a donor supply, aiming to obtain n‐type hBN. The highest S‐atom doping level in hBN has been obtained by using CVD and high‐temperature doping technology. The achievement of n‐type conductive hBN film makes the formation of hBN‐based p‐n junctions possible.

### 2.1 Calculated Electronic Property of Multilayer S‐Doped hBN

**Figure 2:**
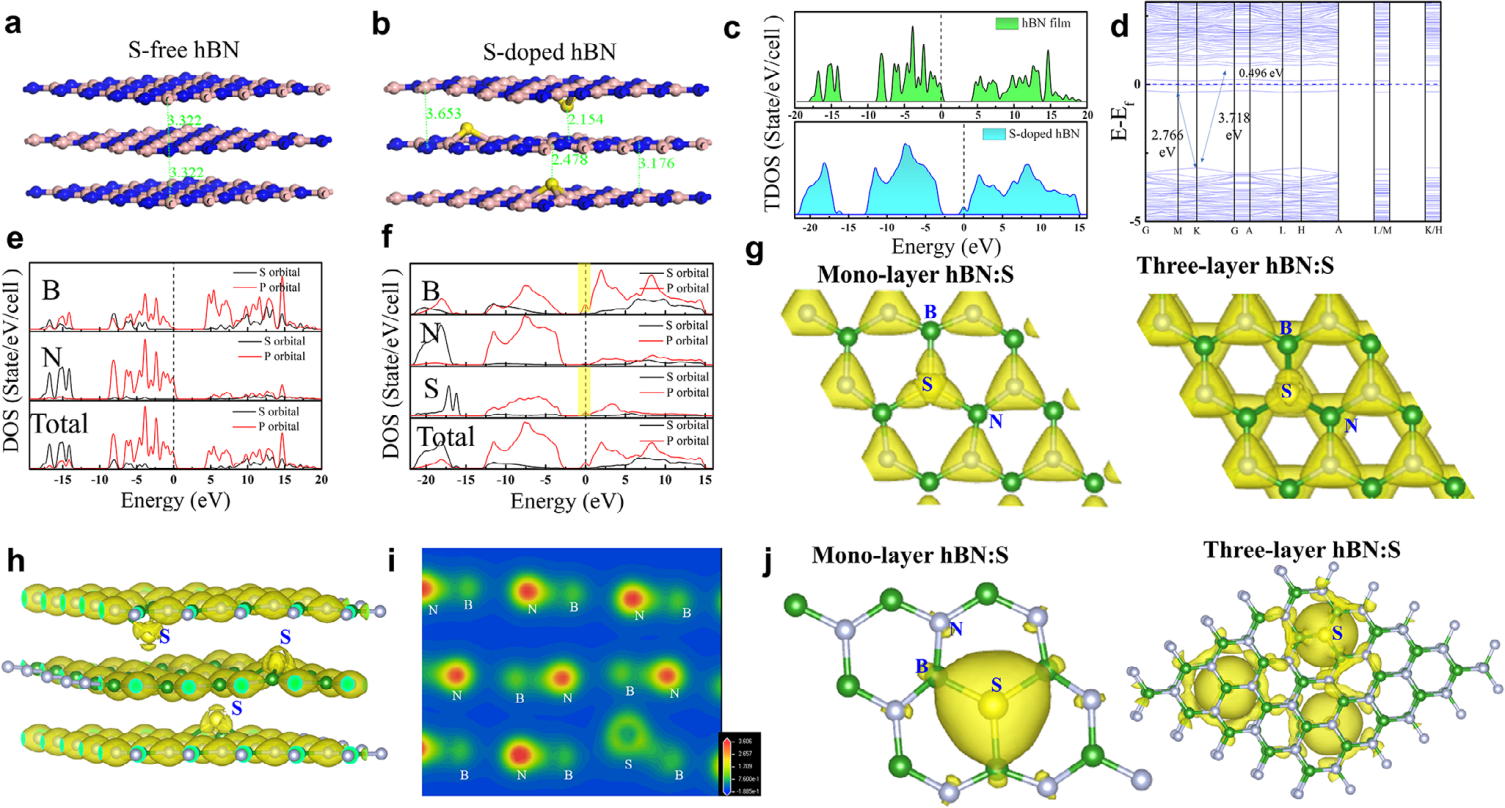
Multilayer structure induced electron delocalization property. a,b) Simulated atomic structure of three layers hBN and S‐doped hBN in our calculation. c) Total density‐of‐states (TDOS) for hBN and hBN: S. d) Electronic band structure of hBN:S using GGA+D2 functional. e) Partial density‐of‐states (PDOS) for h‐BN. f) Partial density‐of‐states (PDOS) for h‐BN: S. g) Iso‐surface plot of charge density with top‐view for mono‐layer and three‐layer hBN:S, iso‐surface value is set to 0.8 electron Å^−3^. h) Iso‐surface plot of charge density with side‐view for three‐layer hBN: S. i) The 2D averaged electron density over the *Z*‐axis for three‐layer hBN: S. j) S‐substitution charge density differences for mono‐layer and three‐layer hBN:S, iso‐surface value is set to 0.11 electron Å^−3^.

Both undoped and S‐doped hBN (hBN:S) films have been comparatively studied by a DFT calculation, in which all the geometry structures are optimized until residual interatomic force is below 0.03 eV Å^−1^. The DFT+D2 was used to describe the van der Waals (vdw) interaction between layers. A layer convergence test was set up, and the convergence threshold for the three‐layer structure was below 0.001 eV atom^−1^, which is capable of representing a multilayer structure. Primarily, the calculated band gaps of a monolayer hBN structure and a three‐layer h‐BN structure are illustrated in Figure S1 (Supporting Information), showing a transition from a direct bandgap (e.g., 4.639 eV) structure to an indirect bandgap (4.379 eV) structure with increasing the number of layers. Hence, the electrical properties of hBN materials depend sensitively on dimensionality, ascribing to an enhancement in the in‐plane overlap among electron and molecular orbitals energy levels. To eliminate any effects due to a change in dimensionality, undoped hBN and hBN:S samples both with a three‐layer structure are selected for the calculation, aiming to investigate the influence of S‐doping on the electrical properties of hBN. As shown in **Figure** **2**a, hBN shows an interlayer space of 3.322 Å and a B─N bond length of 1.448 Å under fully relaxed cases. For a multilayer hBN:S model (Figure 2b), one of the N atoms is substituted by an S atom in each layer, leading to the S component fraction of 4.17%. Although the multilayer hBN:S still remains a 2D layered structure, the substituted S‐atoms are protruded into the 2D plane due to the larger atom radius. The interlayer space decreased to 3.176 Å once the S atoms were protruding in the same direction. In contrast, the interlayer space is increased to 3.653 Å for a reverse protrusion direction of S atoms. The S‐atoms in hBN film drive the change in interlayer distance and should be dependent on the S concentration. The bond length of S─B bonds is elongated to 1.876 Å (see Figure S2, Supporting Information), resulting in a weaker polar covalent bond characteristic than the B─N bonds.

The total density‐of‐states (TDOS) of three‐layered hBN and hBN:S have been calculated as shown in Figure 2c. It can be noted that the *E*_Fermi_ of hBN is located at the edge of the valence band, while the conduction band edge of hBN:S is close to *E*_Fermi_. The results indicate that undoped hBN prefers to show a p‐type conductivity, while hBN:S tends to exhibit n‐type conductivity. The band structure of three‐layered hBN:S has been calculated as shown in Figure 2d, aiming to further determine its conductivity type. It turns out that the S atoms introduce an impurity energy level around the E_Fermi_ and separate the band gap into two parts, one at 0.496 eV below the conduction band minimum (CBM) and another at 2.766 eV above the valence band maximum (VBM). Because the impurity energy level is close to the CBM, the S atoms can be regarded as an efficient n‐type dopant source in hBN. The partial density of states (PDOS) of three‐layered hBN is shown in Figure 2e, indicating that the main contribution to the valence band edge comes from the N 2p orbital, while the conduction band edge is mainly attributed to the B 2p orbital. The PDOS of three‐layered hBN:S has been calculated to understand the orbital interaction between impurity atoms and host atoms (Figure 2f). It is worth noting that the valence band edge originates from the N 2p orbital, while at the conduction band edge, there is a large overlap between the states of the B 2p and S 3p orbitals. The result indicates that there is a strong P‐P orbital coupling between the S 3p orbital and the neighboring B 2p orbital in hBN: S. The S atom will provide a lone pair of electrons and an additional electron during the coupling process, introducing the donor impurity energy levels.

Furthermore, the electrical properties of hBN:S can also be affected by dimensionality. Figure S3a (Supporting Information) shows an optimized atomic structure of monolayer hBN:S, where sulfur atoms are fixed at the plane. The S─B bond length of 1.719 Å in a monolayer case is larger than that of B─N bonds but shorter than that of S─B bonds in a three‐layer hBN:S case (1.876 Å). The band structure of monolayer hBN:S is illustrated in Figure S3b (Supporting Information), suggesting an indirect band gap structure with a value of 4.079 eV. It's noteworthy that the three‐layered hBN:S structure shows a decreased band gap (3.718 eV, Figure 2d) compared to the monolayer structure. This is an intriguing property for hBN:S, meaning that an increase in the number of layers can tune its band gap. The reason is due to the larger S─B bond length together with enhanced electron delocalization in a multilayer hBN:S structure.

The charge density distribution of mono‐layer and three‐layer hBN:S has been compared as shown in Figure 2g. Although S atoms have more valence electrons, the electron densities around S atoms are lower than those of N atoms, meaning that the reduced electronegativity of S is favorable for a more significant charge exchange between S atoms and the h‐BN host lattice. In addition, the electron density of the S atoms in the three‐layer hBN:S displays a further decrease, meaning that more electrons are away from the S atoms with increasing the number of layers. To further ascertain the charge distribution, the side‐view charge density distribution of three‐layer hBN:S is illustrated in Figure 2h. The charge density around S‐atoms shows a significant decrease, suggesting strong electron delocalization in the multilayer hBN:S, which is ascribed to the plane structural deformation and the larger S─B bond length. The 2D averaged electron density over the *z*‐axis (Figure 2i) also validates a significant charge depletion at the S atom. The charge density at the interlayer interstitial region almost does not accumulate, thereby revealing there is not a covalent character between interlayer interaction in the multilayer hBN:S system.

The electron localization of the S atoms in hBN:S with the change of layers has been comparatively studied via S‐substitution charge density differences (Figure 2j). This has been obtained by subtracting the S‐vacancy structure electron density from the S‐substituted structure electron density. The calculated electron density distribution of S‐atom in mono‐layer hBN:S shows strong localization. Nevertheless, the electron distribution tends to spread into the region surrounding the S‐atoms and shows an electron delocalization property in three‐layer hBN: S. The deformation charge density has been calculated to investigate the charge transfer within the crystal, as shown in Figure S4 (Supporting Information). In mono‐layer hBN:S (Figure S4a, Supporting Information), electrons are transferred from B‐atom to N‐atoms and a large number of electrons accumulate at the N‐atom due to the strong polar covalent bond of B─N. However, the S‐atom shows an electron depletion rather than electron accumulation. In three‐layer hBN:S (Figure S4b, Supporting Information), the slice plane is suitably shifted downward for the observation of electron transfer (as shown in the inset of Figure S4b, Supporting Information), because of the downward protrusion of the S‐atom. One can observe the electron depletion at the S‐atom, and that the B‐atoms adjacent to the S‐atom displayed an electron accumulation instead of the electron depletion. The results are consistent with the phenomenon that the electrons from S‐atom spread into the surrounding B‐atom, corroborating the stronger electron delocalization property in three‐layer hBN: S. The stronger delocalization behavior in multi‐layer structures is more favorable for S atoms to perform as efficient donor dopants. As a result, the S atoms in multilayer hBN have greater potential to modulate the bandgap, interlayer space, and charge density distribution.

### 2.2 Incorporation Process of S Atoms into hBN Film

**Figure 3:**
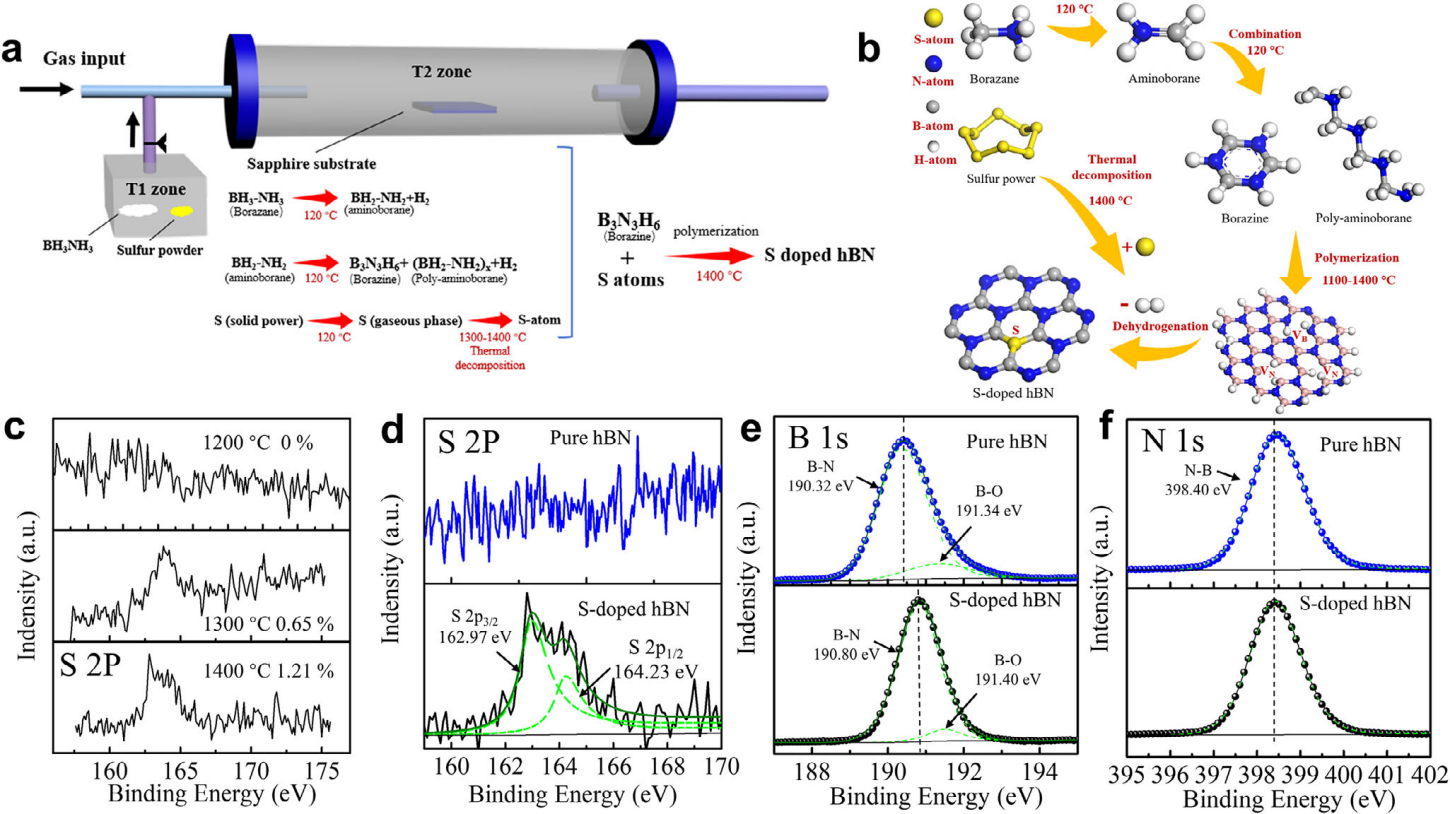
Growth of S‐doped hBN film via LPCVD system. a) Schematic illustration of the LPCVD setup for the S doping in multilayer hBN. b) Reaction pathways for the incorporation of S atoms and the synthesis of hBN:S film. c) XPS spectra of S 2p core‐level of hBN:S film at different growth temperatures. d–f) XPS spectra were measured from d) S 2p, e) B 1s, f) N 1s core‐level of hBN and hBN:S film.

Aiming to eliminate any redundant reaction between the metal substrate and S element during the synthesis of multilayer hBN, sapphire (Al_2_O_3_) has been selected as a substrate. The sapphire substrate can stand at a high temperature such as 1400 °C or above, whereas most of the commonly used metal substrates cannot. An elevated growth temperature helps to meet the formation energy of the S‐substitution defect, leading to an efficient S doping in hBN. A multilayer hBN:S film was prepared by using an LPCVD system as schematically illustrated in **Figure** **3**a. The system consists of two heating zones, where the precursor (borazane powder and sulfur powder) was placed in the TI zone and a sapphire substrate in the T2 zone. Such a system with separated heating zones ensures that only gaseous precursors can be injected into a reaction region (i.e., T2 zone) for growth. The T2 zone, where a sapphire substrate was placed, was heated to 1200–1400 °C under an Ar/N_2_ atmosphere, and then the T1 zone was heated to 120 °C subsequently. Figure 3a also shows an overall precursor decomposition and chemical reaction process within the T1 zone at 120 °C heating. First, the solid borazane (BH_3_NH_3_) was gradually thermally decomposed into aminoborane (BH_2_NH_2_) and gaseous borazine (B_3_N_3_H_6_). In the meantime, the S powders (S8, S4, S2) were transformed to the gaseous phase. Second, the above gaseous decomposition products were carried to the T2 zone by Ar/N_2_ gas flow and a multilayer hBN:S film was then deposited on the sapphire substrate. The relevant reaction processes are illustrated in Figure 3b. Particularly, the initial cross‐link reaction in the T2 zone will lead to the incomplete polymerization of the borazine, coupled with the simultaneous generation of B‐H, N‐H groups, B vacancy (V_B_), and N vacancy (V_N_). The temperature used for the thermal decomposition of gaseous S (S8, S4, S2) was 1473 K (1199.85 °C). Therefore, it is vital to adopt a growth temperature of 1200–1400 °C enabling the thermal decomposition of gaseous S into S atoms. When the cross‐link reaction occurred, the decomposed S atoms were generated in the T2 zone concurrently. Subsequently, the S atoms can be incorporated into the V_N_ with further dehydrogenation, resulting in the synthesis of multilayer hBN:S on sapphire.

### 2.3 Material Characterizations of S‐Doped hBN on Sapphire Substrate

Great attention should be paid to growth temperature, which affects not only the thermal decomposition rate of gaseous S but also the S substitution (S_N_) process. The hBN:S were prepared in the T2 zone at a function of temperature, 1200, 1300, and 1400 °C, aiming to obtain an optimized growth temperature. The other grown conditions for the hBN:S are identical to the epitaxy of the hBN film. X‐ray photoelectron spectroscopy (XPS) is used to quantitatively evaluate the element components and chemical bonding state in a hBN film, where the S 2p spectra of the samples prepared at different growth temperatures were measured as shown in Figure 3c. The S 2p signal was not observed in the sample prepared at 1200 °C, suggesting that almost all of the S gases escape from the reaction region due to a low gaseous S pyrolysis rate at 1200 °C. In that case, it is reasonably hypothesized that 1200 °C is not high enough to ensure the formation of S substitution. Instead, the S 2p signal begins to appear at a doping level of 0.65% when the growth temperature increases to 1300 °C. Further increasing the growth temperature to 1400 °C, the S doping level was verified to be 1.21%, thus confirming that the optimized growth temperature is at least 1400 °C in terms of the formation of S doping due to a high S pyrolysis rate which can be achieved at such a high temperature.

In order to understand the chemical bonding state, the S 2p, B 1s, and N 1s of hBN with or without S doping were carefully fitted as illustrated in Figure 3d–f. In Figure 3d, the S 2p in the hBN:S film was decomposed into two peaks located at 164.23 and 162.97 eV, respectively, corresponding to the S 2p_1/2_ and S 2p_3/2_. The double‐peak characteristic of the S 2p has also been observed in the S powder sample as shown in Figure S5 (Supporting Information). The S 2p signal of S powder was assigned to the core electrons of the S─S bonds, and the fitted double peaks are located at 165.32 (S2p_1/2_) and 164.14 eV (S2p_3/2_). The cleavage of S 2p peaks should be peculiar to the phenomenon of electron spin‐orbit coupling. Based upon the fitted double‐peak of S 2p with a peak intensity ratio of 1:2 (S 2p_1/2_: S 2p_3/2_) in the hBN:S film (Figure 3d), the S atom remains in a single bonding state. The binding energy of S 2p in the hBN:S film shows a reduction compared to the S─S bonds in S powder, suggesting that the S‐atom bonded with the B‐atom (formation of B─S bonds) in the hBN:S film due to the lower electronegativity of B‐atom (2.04) than the S‐atom (2.58). The B 1s spectra of hBN and hBN:S were investigated in Figure 3e, which shows a B─N bond at 190.45 eV and a shoulder peak (191.34 eV) assigned to the B─O bonds in the hBN film. However, the B 1s core electrons of B─N bonds in the hBN:S film are located at 190.80 eV. The higher binding energy of B 1s in hBN:S film should be related to the existence of the B─S bonds since the atomic number of the S element is larger than that of the N element. The position of N 1s peak (398.40 eV, Figure 3f) is almost the same in hBN and hBN:S samples, which elucidates the N‐atoms free from the bonding state with S‐atoms. The elemental ratios of hBN and hBN:S film can also be accurately determined via the XPS results, and the B/N ratios of hBN and hBN:S film are 1.12:1 and 1.03:1, respectively. In hBN:S film, the N‐deficiency was largely alleviated, suggesting the suppression of V_N_ defect in film. The component ratio of the B─O bonds in B content decreased from 14.91% to 8.62% after S doping, where the B─O bond originates from the formation of the B_x_O_y_ complex by binding negative oxygen ions with B interstitial (B_i_) defects. Hence, the S atoms are incorporated into V_N_ and bonded with B atoms, so the S dopants are favorable for the suppression of B_i_ and V_N_ defects in film and may have the capacity for improving the crystallinity.

**Figure 4:**
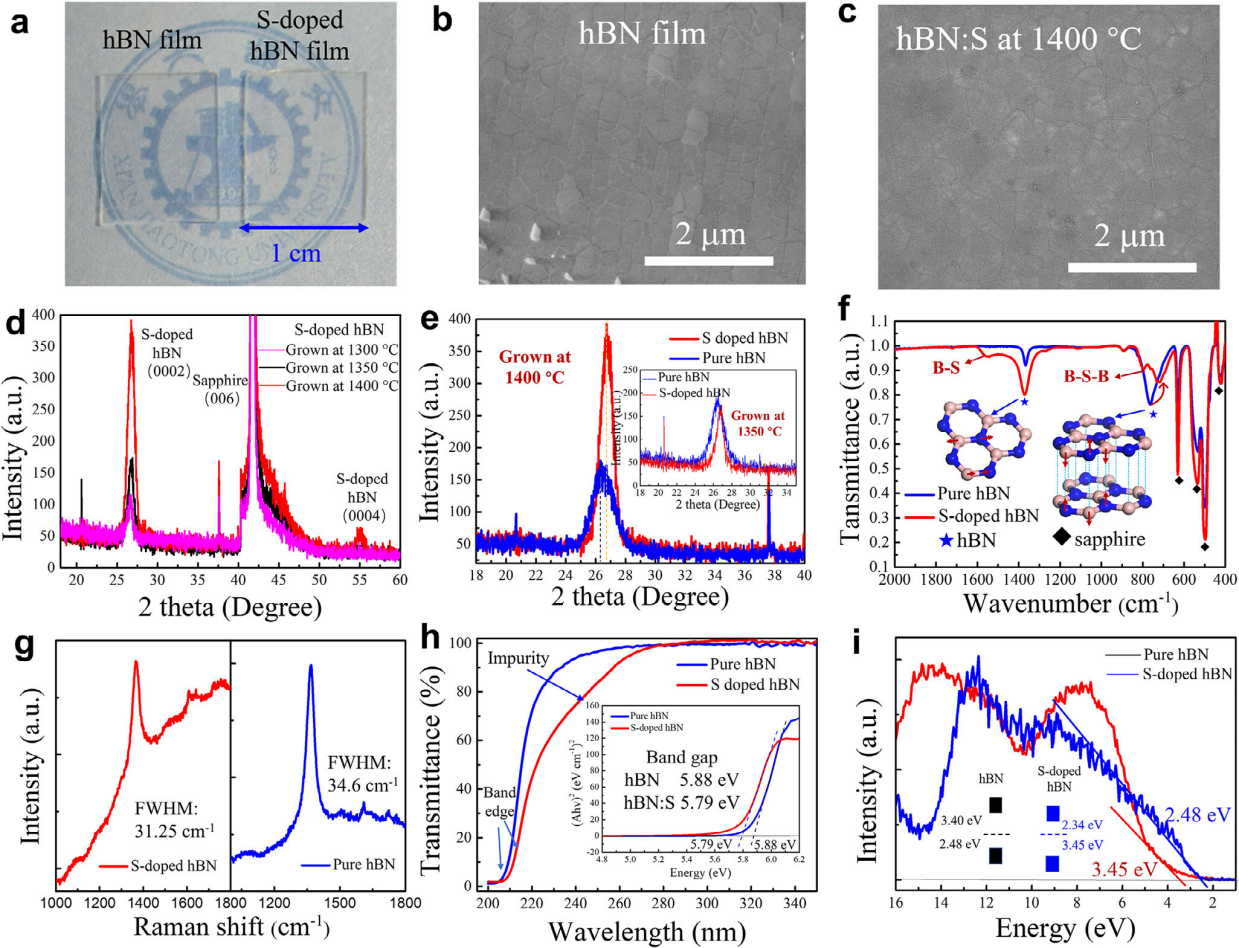
Characterization of S‐doped hBN on sapphire. a) Photograph of hBN and hBN:S film synthesized on a sapphire substrate. b) Top view SEM images of the hBN film on a sapphire substrate. c) Top view SEM images of the hBN: S film synthesized at 1400 °C. d) XRD characterization of hBN:S film grown at 1300, 1350, and 1400 °C on sapphire substrate. e) Comparison of XRD patterns of pure hBN films and hBN:S films at growth temperatures of 1350 and 1400 °C. f) The FTIR spectrum of hBN and hBN:S film on sapphire substrate. g) Raman spectrum of hBN and hBN:S film on sapphire substrate. h) UV–vis absorption spectrum of hBN films and hBN:S films on sapphire substrate. The inset is an optical band gap analysis of hBN films and hBN:S films. i) Valence band spectra of hBN films and hBN:S films. The inset shows the schematic illustration of the band diagrams for pure hBN films and hBN:S films.

Both the as‐grown hBN film and the hBN:S film on sapphire substrates are transparent as confirmed in **Figure** **4**a. Figure 4b shows the scanning electron microscope (SEM) image of the large‐area, continuous hBN film on a sapphire substrate. The hBN on sapphire has a signature honeycomb wrinkled surface morphology, which is similar to those observed in the previous studies. An atomic force microscope (AFM) was used to further confirm the wrinkled topography by unraveling the height undulation of the hBN film in Figure S6 (Supporting Information). The honeycomb wrinkled surface of hBN was further verified in the AFM image, showing a wrinkle height of 1.5–3.1 nm. For the hBN:S film prepared at 1200 °C (Figure S7a, Supporting Information), the honeycomb wrinkles are not observed, suggesting the film still remains a turbulent layer structure, which is proved by the absence of the (0002) diffraction peaks in XRD results (Figure S8, Supporting Information). When the growth temperature was increased up to 1300 °C, the honeycomb wrinkle started to appear and was accompanied by a plethora of cluster particles on the surface (Figure S7b, Supporting Information). The temperature of 1300 °C is not high enough to pyrolyze sulfur gaseous, thereby S atoms cannot be supplied and supersede the N atoms sufficiently. In that case, it is likely that the excessive S precursors just form by‐products above the surface. With further increasing the growth temperature to 1350 °C, one can observe a minority of particles on the surface (Figure S7c, Supporting Information). Once the growth temperature reaches 1400 °C, the film shows a typical honeycomb wrinkled morphology with a clean surface (Figure 4c), indicating a high pyrolysis rate of S precursor and a high S‐atoms incorporation rate into the hBN lattice at 1400 °C. Hence, a high temperature of 1400 °C is necessary for S‐atom substitution doping into hBN film. From the results of top‐view SEM images, the hBN:S film grown at 1400 °C also displays a more distinct surface wrinkle than an undoped hBN film. Therefore, one can extrapolate that the layered structure can be improved after S doping and should behave with a better crystal quality. The energy dispersive spectroscopy mapping of N_K_, B_K_, and S_K_ was detected from a randomly selected area of the samples (Figure S9, Supporting Information), where the S elements were evenly distributed within the hBN film.

The crystallinity of hBN:S films was examined by using X‐ray diffraction (XRD). Figure 4d shows the XRD data of the hBN:S samples grown at different temperatures (1300, 1350, and 1400 °C). The (0002) diffraction peaks exhibit a tendency to appear in the hBN:S film prepared at 1300 °C. The peak intensity of XRD is on behalf of the lattice plane content and orientation degree, that is, the crystallinity of the crystal. The (0002) diffraction peak intensity shows a tendency to increase with the increase of the growth temperature. The FWHM of the XRD peak presents the degree of ordering of the lattice plane arrangement, with the values of 0.90°, 0.89°, and 0.92° at 1300, 1350, and 1400 °C, showing a negligible change. Hence, the higher the growth temperature, the greater the crystallinity of the hBN:S films. The position of the diffraction peak (26.72°) almost remains unchanged in spite of using different growth temperatures. Therefore, it can be concluded that the growth temperature was one of the key parameters for preparing the hBN:S film. The XRD data of an undoped hBN sample and a hBN:S sample grown at 1350 and 1400 °C are provided in Figure 4e. It's worth noting that the experiments compared S‐doped hBN to pure hBN in this work all grown under identical conditions. The full width at half maxima (FWHM) of hBN film was 1.67° prepared at 1350 and 1400 °C. The full width at half maxima (FWHM) of hBN film prepared at 1350 and 1400 °C was 1.67°. The FWHM of hBN:S narrows down to 0.89° and 0.92° at 1350 and 1400 °C. Comparison of their FWHM indicates an impressive improvement in the crystallinity of the hBN:S film compared with that of hBN prepared at 1400 °C, and the crystallinity of the undoped hBN also was inferior to the hBN:S when grown at 1350 °C. (the inset of Figure 4e). The improved crystal quality of the hBN:S film is on account of the suppression of V_N_ and V_B_ via the generation of S_N_, which aligns with the XPS analysis. Furthermore, the diffraction peak located at 26.43° for the undoped hBN film exhibits a left shift compared with the hBN:S film (26.72°). The XRD peak position of 26.43° in the hBN film and 26.72° in the hBN:S film corresponds to an interlayer distance of 3.37 and 3.33 Å, respectively. The decrease of interlayer stacking distances with S‐doping in hBN nanosheet (h‐BNNS) powder has been observed in previous work,^[^ ^46^ ^]^ and our work demonstrates for the first time that the epitaxial growth of multi‐layer hBN:S film has the same trend. The changed interlayer distance after S doping is also consistent with the DFT analysis, that is, the structural distortion induced by the S‐atom leads to the change of interlayer spacing.

Fourier transform infrared spectroscopy (FTIR) has been measured on the undoped hBN and hBN:S as shown in Figure 4f. Two characteristic absorption peaks of the undoped hBN are located at 1367.5 and 765.7 cm^−1^, which are assigned to the in‐plane B‐N stretch mode and the out‐of‐plane B‐N‐B bending vibration mode. For the hBN:S film, the positions of all absorption peaks are identical to the undoped hBN except for the 725.2 cm^−1^ absorption peak (corresponding to the B‐N‐B bending vibration mode of 765.7 cm^−1^ in pure hBN). The change in the interlayer distance can be manifested in the change of out‐of‐plane B‐N‐B bending vibration frequency, and thus the rightward shift of the low‐wavelength absorption peaks in the hBN:S film is observed. There are also two apparent shoulders at 1553.7 and 800.4 cm^−1^ in hBN:S film, correlating with the in‐plane B‐S stretch and out‐of‐plane B‐S‐B bending vibration after S‐substitution doping.^[^ ^47^ ^]^ Moreover, the intensity of the high‐wavenumber absorption peaks of the hBN:S film outpaces that of the low‐wavenumber absorption peaks, which is opposite to the undoped hBN film. The B‐N‐B bending vibration depends largely on the *π* bonding within the 2D plane to be broken, therefore, the noticeably weaker intensity of the low‐wavenumber peak in hBN:S film comes down to the suppression of the vacancy defects after S‐substitution doping. The Raman spectra of the hBN:S film (Figure 4g) displayed an in‐plane E_2_ _g_ vibration mode of hexagonal B─N bonds at 1366.8 cm^−1^,^[^ ^41^ ^]^ and therefore the hBN:S film was determined to remain the hexagonal crystal structure. The FWHM of hBN:S (31.25 cm^−1^) was narrower than that of the undoped hBN (34.6 cm^−1^), thereby indicating that the crystal quality of the hBN:S film is better than that of the undoped hBN. The fluorescence observed from the hBN:S film is highly likely associated with the existence of S‐elements in the film.

Figure 4h displays the UV–vis absorption spectra of the undoped and the S‐doped hBN on sapphire, indicating that the hBN:S film has a significant red‐shift in band‐edge absorption. Additionally, an absorption shoulder at 240–270 nm appears in the h‐BN:S film, which should be attributed to the S impurities‐assisted defect absorption. The inset of Figure 4h provides a summary for the measured bandgaps of the samples, that is, 5.88 eV for the undoped hBN and 5.79 eV for the hBN:S, respectively. The decreased band gap aligns with the calculated electronic band structure of multi‐layer hBN: S. Figure 4i shows the X‐ray photoelectron valence band (VB) spectra of both the undoped hBN and the hBN: S. The valance band maxima (VBM) of films can be determined by linearly extrapolating the leading edge of the valance band spectra. So, the VBM of undoped hBN was determined to be 2.65 eV below the fermi energy level, and the related band structure is shown in the inset of Figure 4i. The weak P‐type conductivity in pure hBN relies largely on the generation of V_B_, besides, the B_i_ is feasible to combine with negative oxygen ions to form B_x_O_y_ complexes,^[^ ^40^ ^]^ thereby stripping electrons from the hBN system, as corroborated with the generation of B─O bonds in XPS results (Figure 3e, undoped hBN). The calculated VBM of hBN:S film was 3.45 eV below the E_Fermi_, and the band structure in the inset shows a typical n‐type semiconductor conductivity. The content of B_x_O_y_ complexes was reduced in hBN:S film (Figure 3e, hBN:S), which was in favor of the suppression of V_B_ and B_i_. Therefore, the VI‐group S‐atom substitutional doped into hBN film has the capacity for defect compensation and also contributes additional electrons to improve the n‐type conductivity. The large‐scale high‐crystallinity hBN:S film also holds the aptitude for integration with existing semiconductor processes, as we proved in previous work.^[^ ^40^, ^41^ ^]^ For instance, the films have a high‐temperature tolerance of over 1100 °C, which is compatible with semiconductor epitaxial processes where other epilayers can be grown directly on top of it. The large‐scaled intact exfoliation of hBN from sapphire substrate via a contamination‐free mechanical exfoliation process also manifesting the capability for the choice of arbitrary substrates.

### 2.4 Ionization Energy Analysis of S Impurity Energy Level

**Figure 5:**
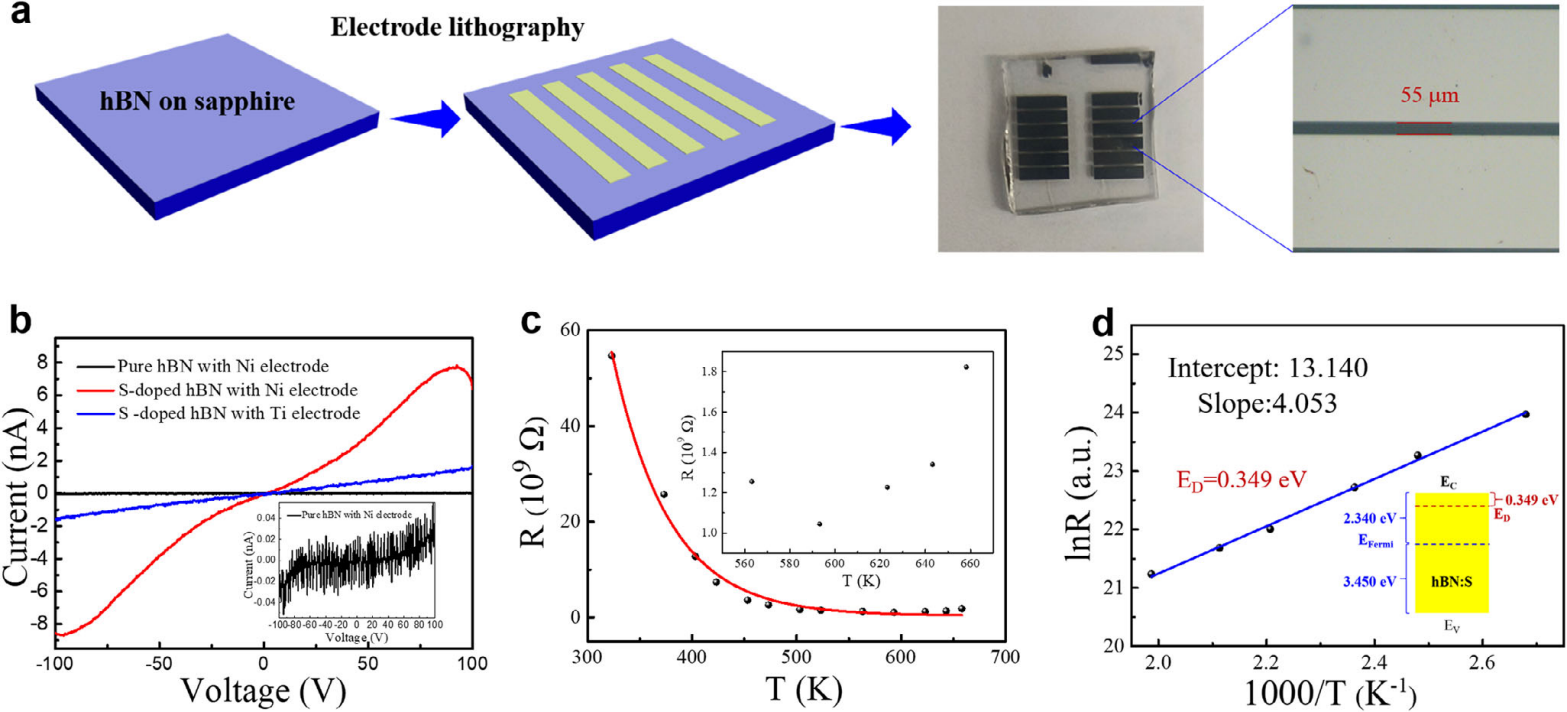
Ionization energy analysis of S impurity energy level. a) Schematic diagram and photograph of the electrode pads on the film. b) The *I–V* curve of the hBN:S film with Ni and Ti electrodes. The inset is the *I–V* curve of the pure hBN film with Ni electrode. c) The temperature dependence of the dark resistance of an h‐BN:S sample from 323–658 K. The inset is the local enlargement view at 563–658 K. d) The Arrhenius plot and the fitted curve of the temperature‐dependent resistance in hBN:S film from 373–503 K. The inset is the fitted value and the band diagrams of the hBN:S film.

In order to investigate the electrical properties of the hBN:S films, the electrode pads with a 55 µm spacing were deposited on the film using a photolithography technique (**Figure** **5**a). *I–V* measurements were conducted on the undoped hBN as illustrated in the inset of Figure 5b. A voltage range of −100–100 V was used for the measurements, showing conclusively that a highly insulating feature for hBN film with a current of −30–30 pA. This implies that a hBN film grown on sapphire has great potential for being used as a dielectric layer. In Figure 5b, the hBN:S film with a Ni electrode exhibits a striking increase in surface current with a result of 8.4 nA under 100 V external voltage. The improved conductivity of hBN:S film depends on the efficient excitation of the S donor impurity, resulting in a higher carrier density. On the other hand, a Schottky contact between Ni and hBN:S film can also be confirmed from the *I–V* curve, and the schematic layout of the formation of the Schottky potential barrier at the interface is illustrated in Figure S10 (Supporting Information). Due to the high work function of Ni electrode (5.15 eV), the depopulation of electrons occurred at the hBN:S side, and then an upward potential barrier on the hBN:S side is formed. In turn, the n‐type conductivity can be reconfirmed for the hBN:S film in light of the Schottky contact property. However, an ohmic contact property with a perfectly linear *I–V* curve was demonstrated by using the Ti electrode deposited onto the hBN:S film (Figure 5b). As an illustration of the band alignment of Ti/hBN: S in Figure S11 (Supporting Information), a magnitude of electrons accumulated on the hBN:S side should be adequate for the formation of ohmic contact, because of the low work function of the Ti electrode (4.33 eV). The lower surface current in the Ti/hBN:S sample under 100 V voltage (1.6 nA) than that of the Ni/hBN:S sample should be accounted for by the fact that the higher resistivity index of Ti metal.

*I–V* curves were also measured using a variable‐temperature *I–V* test platform (Figure S12, Supporting Information) under various temperatures with a range of 30–385 °C, where intrinsic thermal excitation can be safely ignored in this low‐temperature range. As shown in Figure S13 (Supporting Information), the surface current of hBN:S with a Ti electrode increases from 1.6 to 90 nA under 100 V with increasing temperature (30–385 °C), which is due to the thermal activation of the S impurity allowing for an improvement in the carrier density and the conductivity of the hBN:S film. There is also an abnormal phenomenon that the current shows a decrease since the start of the 350 °C. The temperature‐dependent resistance (*R*) result in Figure 5c and local enlargement of 563–658 K in the inset both show an increased resistance at 350 °C (623.15 K). So, it can be determined that the strong ionization region of S impurities is reached at 350 °C, where lattice vibrational scattering plays a major role. At the range of 50–320 °C (323.15–593.15 K), the S‐dopants in the hBN:S films are in the weak ionization region, and the change of carrier mobility (*μ*) could be negligible due to the low doping concentration of S‐atoms. Therefore, the relationship between resistance and temperature in a range of 50–320 °C can be described using an equation provided below.^[^ ^24^ ^]^

(1) $$R=R_{0}\exp\left(\frac{\Delta E_{D}}{k_{0}T}\right)$$

where the ∆*E*_D_ denotes the ionization energy of S impurities energy level (E_C_–E_D_), the k_0_ represents Boltzmann's constant, and the *R*_0_ is a constant. The above equation was further elaborated in Figure S13 (Supporting Information). The fitting plots of the temperature‐dependent resistance (*R*) in Figure 5c certainly displayed the same tendency with the equation over a range of 50 to 320 °C. Subsequently, the value of ∆*E*_D_ of the S‐impurity energy level can be deduced by transforming and fitting Equation (1) using an Arrhenius plot as the following equation.^[^ ^31^, ^35^ ^]^

(2) $$\ln R=\ln R_{0}+\frac{\Delta E_{D}}{1000k_{0}}\frac{1000}{T}$$

The linearly fitted plot of ln (*R*) versus 1000/*T* was shown in Figure 5d, where the value of intercept and slope were fitted to be 13.140 and 4.053, respectively. The calculated value of ∆*E*_D_ of the S‐impurity energy level is 0.349 eV. The diagrams of the position of *E*_D_ and *E*_Fermi_ in hBN:S were illustrated in the inset of Figure 5d.

### 2.5 hBN: Mg/hBN:S Homojunction for Deep‐UV Light‐Emitting

A vertically stacked n‐hBN/p‐hBN homo‐structure was obtained in this work, where the p‐hBN was achieved by doping magnesium (Mg) elements into the hBN film. The elemental Mg was the most propitious p‐type dopant in an hBN film, and the Mg_2_N_3_ as an Mg source was widely used in previous work.^[^ ^29^, ^30^ ^]^ In this study, the Mg‐doped hBN film (hBN: Mg) was synthesized on a Cu substrate and the XPS spectrum (including B 1s, N 1s, and Mg_Auger_) of hBN: Mg was shown in Figure S14 (Supporting Information). The measured binding energies of B1s and N1s (Figure S14a,b, Supporting Information) in hBN: Mg film were 190.4 and 398.2 eV, which are in agreement with the B─N bonds. The Mg_Auger_ peak (Figure S14c, Supporting Information) of hBN: Mg film appeared at 305.5 eV, suggesting the successful incorporation of Mg atoms into hBN and the generation of Mg─N bonds.^[^ ^29^, ^30^ ^]^ In Figure S15a (Supporting Information), the XPS full spectra of hBN: Mg film were measured to estimate the concentration of Mg element. In contrast to the XPS full spectra of pure hBN (Figure S15b, Supporting Information), an additional Mg 1s (1303.7 eV) and Mg_Auger_ (305.5 eV) signal can be observed in hBN: Mg film. The Mg 1s peak was selected to determine the concentration, and the substitution concentration of Mg is 3.27%. The synthesized hBN: Mg film on Cu foil was transferred onto a sapphire substrate with the conventional PMMA‐assisted liquid transfer,^[^ ^48^ ^]^ and then the UV–vis spectrum was measured in Figure S16 (Supporting Information). One can see that the absorption edge of hBN: Mg film is still below 220 nm, and the band gap value was determined to be 5.94 eV (the inset of Figure S16, Supporting Information). From the valence band spectra and band diagram of hBN: Mg film (Figure S17, Supporting Information and its inset), it can be concluded that the Mg dopants were quite at ease with efficient p‐type doping in hBN with the VBM of 2.10 eV below the *E*_Fermi_. The *I–V* curve of hBN: Mg film using Ni and Ti as electrodes is shown in Figure S18 (Supporting Information). The conductivity of the hBN: Mg film shows a great improvement, which is confirmed by an increase in surface current. Furthermore, compared to the Ti electrode, the easiness of forming an ohmic contact with the Ni electrode can also confirm that the hBN: Mg film holds a P‐type conductivity.

**Figure 6:**
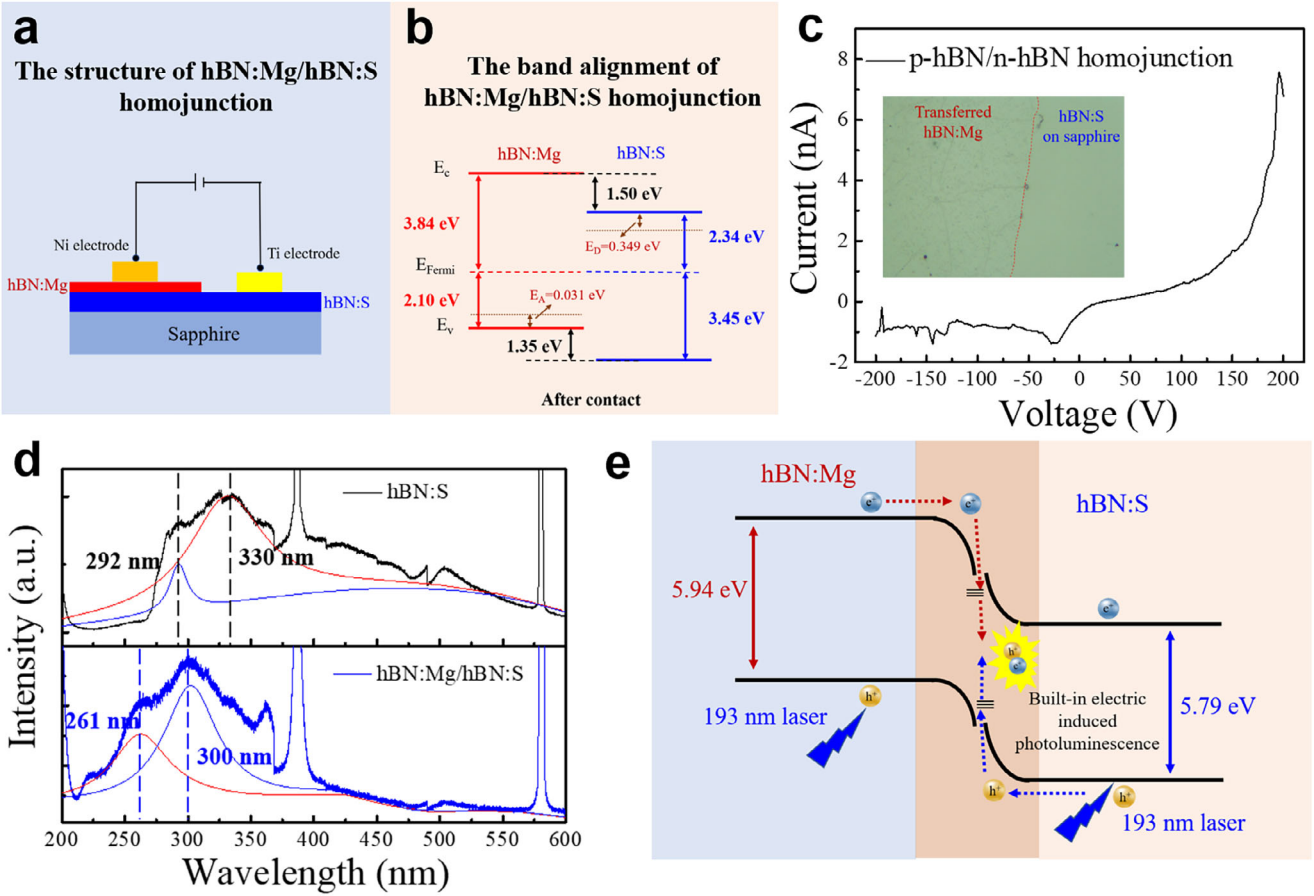
Photoluminescence properties based on hBN: Mg/hBN:S homojunction. a) The schematic of the p‐n homojunction based on hBN: Mg/hBN: S. b) The band diagram of the p‐hBN: Mg/n‐hBN:S homojunction. c) *I–V* curves of the p‐hBN/n‐hBN junction based on hBN: Mg and hBN:S film. The inset is the photograph of the transfer‐constructed p‐n junction. d) The PL spectra of hBN:S film and hBN:S/hBN: Mg homojunction via direct epitaxy. e) The schematic presentation of the photoluminescence process within hBN:S/hBN: Mg homojunction.

The p‐n junction was fabricated by transferring the hBN: Mg film onto the surface of hBN:S film, which subsequently underwent a baking treatment (300 °C for 30 min) aiming to improve the interface contact quality. The structure of the p‐n homojunction used in this work was sketched in **Figure** **6**a, where the Ni electrode and Ti electrode were deposited on the hBN: Mg and hBN:S respectively to form the ohmic contact. The *E*_A_ value of Mg impurity energy level in hBN: Mg film has been calculated to be 31 meV in previous work.^[^ ^31^ ^]^ Hence, the detailed band configuration of the hBN: Mg/hBN:S homojunction was concluded in Figure 6b and shows a staggered band alignment after contact. The staggered band alignment and large band edge offset favor the application of hBN: Mg/hBN:S homojunction as a light‐emitting diode. The photograph of the p‐hBN/n‐hBN (hBN: Mg/hBN:S) junction prepared by transfer technology is shown in the inset of Figure 6c, where wrinkles can be observed at the hBN: Mg film. Photograph of other positions is tested to reconfirm the regular generation of the wrinkles in hBN: Mg film after being transferred onto the hBN:S surface, and the wrinkled surface can also be observed (Figure S19, Supporting Information). In Figure 6c, the *I–V* curves of the hBN: Mg/hBN:S homojunction displayed a diode rectification behavior via simple transfer technology, signifying the generation of a built‐in field due to the contrary majority carriers and carriers diffusion within the interface. The easiness of the generation of space charge region within hBN: Mg/hBN:S is on account of the same hexagonal crystal structure with low junction formation energy, however, the wrinkles at hBN: Mg layer inevitably degrade the interface contact quality and will induce severe nonradiative recombination at the interface.

The hBN: Mg film was also attempted to grow directly on a hBN: S/sapphire template, attempting to pursue a high interfacial quality for hBN: Mg/hBN:S homojunction. Epitaxial growth of hBN: Mg film was carried out by using the LPCVD system. An entire coverage of hBN: Mg film over the surface of the hBN: S/sapphire template was confirmed, where the XPS spectra of randomly selected regions exhibited a notable Mg_Auger_ peak (Figure S20, Supporting Information). The close interface contact of as‐synthesized hBN: Mg/hBN:S homojunction circumvents the severe nonradiative recombination at the interface, which opens up an avenue to explore the luminescence property of the homojunction by using PL technology. The PL measurement of hBN:S film and hBN: Mg/hBN:S homojunction was fitted and illustrated in Figure 6d. In hBN systems, the unique optical property is that the defects‐related luminescence shows stronger intensity than the intrinsic luminescence, including sheet deformation, stacking faults, and vacancy‐impurity defects.^[^ ^6^, ^7^, ^42^, ^43^ ^]^ It has been proved that the D lines and S lines (located at 5.4–5.9 eV) of hBN in PL measurement tend to all disappeared at room temperature (280–300 K),^[^ ^49^, ^50^, ^51^ ^]^ and the PL tests in this work were all performed at room temperature (300 K). The obvious modulation observed on the top of the broadband suggests strong evidence that the central luminescence of hBN:S film comes from defect transitions involving doped S atoms,^[^ ^42^, ^51^ ^]^ and the impurity atoms also hold the affect to inhibit the intrinsic transition.^[^ ^10^, ^51^ ^]^ Therefore, the fitted photoluminescence peak of hBN:S film at 330 and 292 nm are both induced from the donor–acceptor pairs transition involved with the localized acceptor complex center, because of the doping and polycrystalline property of films. Two fitted peaks in the PL spectra are due to the different layers stacking configurations in hBN:S film. Once below the wavelength of 282 nm, the luminescence intensity decreases rapidly and cuts off completely at ≈270 nm, showing a limited luminescence capability of individual hBN:S film at the DUV range. However, the central PL peak of hBN: Mg/hBN:S homojunction anomalously blue‐shifts to 300 nm, irrespective of the front excitation or back excitation (Figure 6d; Figure S21, Supporting Information). Particularly, the short‐wavelength fitted peak to the left of the central PL peak also blueshifts to 261 nm in homojunction. Hence, the hBN: Mg/hBN:S homojunction is endowed with the capability in the DUV light‐emitting. In Figure S22 (Supporting Information), the hBN: Mg film shows the same PL peak position and cutoff edge with the hBN:S film due to the similar donor–acceptor pairs transition luminescence process, however, the notable weaker PL intensity is on account of the lower crystallinity of hBN: Mg film. It has been proved that the doping level we achieved can be able to form a space charge region within the homojunction, and the anomalous blue shift of the luminescence peak of the homojunction luminescence is caused by the space charge region.

The photoluminescence process of the hBN: Mg/hBN:S homojunction driven by the built‐in electric field was schematically illustrated in Figure 6e. Photogenerated carriers are excited in each layer of the homojunction under laser excitation at a wavelength of 193 nm. Photogenerated electrons in hBN: Mg and the photogenerated holes in hBN: Mg drift into the space charge region with the aid of the built‐in electric field, and then undergo radiative recombination to release photons. Since the doped multilayer hBN film is an indirect bandgap material with sub‐energy levels between the bandgaps, photogenerated carriers drifting into the space charge region will first transit to the sub‐energy levels, followed by radiative recombination luminescence, and thus luminescence located at a blue‐shifted wavelength appears in the PL test. Optical tests verify the potential of the homojunction for deep‐UV luminescence, and also its ability to be further extended into the short wavelength region. This work surmounts the limitation of n‐type conductivity in hBN by substitutional doping of the S‐atoms, and the construction of hBN: Mg/hBN:S homojunction is forecast to be a novel direction of 2D ultrawide band gap Vdw junction structure. The hBN: Mg/hBN:S homojunction also holds great potential application in the field of deep‐UV photonics, and provides the feasibility of device structure design.

## 3 Conclusion

In conclusion, a large‐scale, multilayered n‐type h‐BN film has been obtained on a sapphire substrate via Sulfur doping by an LPCVD system. S‐doped hBN (hBN:S) was calculated and studied by using DFT, demonstrating the generation of shallow donor impurity levels due to the orbital coupling between S 3p and B 2p. The influence of dimensionality on the electrical properties of hBN:S was calculated and analyzed. The calculation results confirmed that the multi‐layer hBN:S performs a better electron delocalization property, and a great potential for tuning bandgap, interlayer space, and charge density distribution. Subsequently, the growth temperature was optimized for the experimental synthesis of multilayer hBN:S films on sapphire substrates, and the optimized temperature is confirmed to be 1400 °C based on XPS and SEM results. The S content of 1.21% in hBN:S was obtained. The substitutional S_N_ is expected to mitigate the B_i_ and V_N_ within the film, resulting in an improved crystalline quality. The improved crystallinity and the reduced interlayer distances in the hBN:S film compared with undoped hBN film have been verified by XRD results. This work also demonstrated the n‐type conductivity of hBN:S film with a considerably enhanced in‐plane current (1.6 nA with Ti electrode and 8.4 nA with Ni electrode) via valence spectra measurements and Schottky/Ohmic contact analysis. The ∆*E*_D_ of the S‐impurity energy level in hBN:S is calculated to be 0.349 eV. The p‐n homojunction constructed from hBN: Mg/hBN:S displayed a decent diode behavior and deep‐UV photoluminescence, which can broaden the application of optoelectronic and electronic devices using the approach stated in this study.

## 4 Experimental Section

### Theoretical Calculations

Cambridge Sequential Total Energy Package (CASTEP) was used to perform calculations based on a density functional theory (DFT). The exchange and correlation effects were described by using the generalized gradient approximations (GGA) of Perdew, Burke, and Ernzerhof (PBE). The vdw interaction for the three‐layer h‐BN and the hBN:S was described by DFT+D2. Core electrons were replaced by ultra‐soft pseudopotential in the calculation, where the B(2s^2^2p^1^), N(2s^2^2p^3^), and S(3s^2^3p^4^) were considered as valence electron configurations. All the structures were assumed to be fully relaxed in an optimized scenario, where the residual interatomic force was less than 0.03 eV Å^−1^. The Brillouin zone was sampled with the equivalent of 7 × 7 × 1 k‐points for calculation. The cut‐off of 550 eV was used for expanding the electronic wavefunctions and a vacuum region of 15 Å along with the *z*‐axis was established to separate the periodic replicas. During the charge density and density difference calculation, the iso‐surface value was set to 0.8 electron Å^−3^ and 0.11 electron Å^−3^.

### Epitaxial Growth of hBN Film

hBN, hBN:S, and hBN: Mg films were prepared by using an LPCVD system (GSL‐1600 × 220 V φ60 mm) featuring two heating zones. A sapphire substrate (a thickness of 430 µm) was selected for the growth of hBN and hBN:S films. A Cu foil (Alfa Aesar, 25 µm) was used as a substrate for the growth of hBN: Mg film. Borazane (BH_3_‐NH_3_, 97%, Sigma‐Aldrich), sulfur powder (99.98%, Sigma‐Aldrich), and magnesium nitride (99.5%, Mg_2_N_3_, Macklin) served as precursors to supply B/N source, S source, and Mg source, respectively. For the epitaxial growth of hBN films on sapphire, the sapphire which was placed in the T1 zone was heated to 1400 °C under an Ar/N_2_ atmosphere (30 sccm:10 sccm). Subsequently, the borazane (40 mg) was heated to 120 °C in the T2 zone, and the gaseous decomposition was carried out in the T1 zone under Ar/N_2_ flow (60 sccm:20 sccm). The parameters of growth pressure and growth time were set to be 1 torr and 40 min, respectively.

### Epitaxial Growth of hBN:S and hBN: Mg Film

For the epitaxial growth of hBN:S film on sapphire, the sapphire substrate in the T1 zone was heated to 1200, 1300, 1350, 1400 °C for each case. Subsequently, the S powder (40 mg) in the T2 zone was simultaneously heated with borazane at 120 °C. The other parameters were identical to those for the growth of hBN film. For the epitaxial growth of hBN: Mg film on a Cu foil, the Cu foil was first heated to 1050 °C. Second, the Mg_2_N_3_ as an Mg source also was thermal decomposition within the T1 zone at 1050 °C. At the same time, the borazane was supplied for the growth of the hBN: Mg film on a Cu foil. The growth pressure and the growth time used were 500 mtorr and 15 min, respectively.

### Fabrication of hBN:Mg/hBN:S Homojunction—Transfer of hBN:Mg onto hBN:S/Sapphire

The matured polymethyl methacrylate (PMMA) assisted liquid phase exfoliation^[^ ^48^ ^]^ was used to transfer the hBN: Mg film (Cu foil) onto the hBN: S/sapphire. First, the surface of the hBN: Mg film was spin‐coated with PMMA. The PMMA/h‐BN: Mg/Cu was immersed in an aqueous (NH_4_)_2_S_2_O_8_ solution to remove the Cu foil. After 6 h, the Cu foil was completely corroded away and then the PMMA/hBN: Mg was transferred onto the hBN: S/sapphire template. Subsequently, the PMMA was removed by acetone to achieve a hBN: Mg/hBN:S homojunction. Finally, 30 min baking at 300 °C under an Ar atmosphere was conducted in order to improve the interface quality.

### Fabrication of hBN: Mg/hBN:S Homojunction—Direct Growth of a hBN: Mg Film on a hBN: S/Sapphire Template

A hBN: S/sapphire template which was placed in the T1 zone was heated to 1100 °C. Mg_2_N_3_ and borazane were then supplied simultaneously to grow a hBN: Mg film on the hBN: S/sapphire template. The growth pressure and the growth time were chosen to be 500 mtorr and 15 min, respectively. Both hBN:S and hBN: Mg film were selected with a thickness of 8 nm.

### Characterization

X‐ray Photoelectron Spectroscopy (XPS) and Valence band spectra were measured using a Kratos Axis Ultra DLD spectrometer (Manchester, UK) equipped with a monochromatic Al Kα source operated at 150 W. The experiment data of XPS were fitted using a Voigt function and are calibrated using C 1s peak at 284.8 eV as a reference. Scanning electron microscope (SEM, Quanta 250FEG) was conducted to examine the surface morphology of the samples. X‐ray diffraction (XRD) measurements were conducted using X'Pert PRO (Almelo, Netherlands). Fourier transform infrared spectroscopy (FTIR) measurements were carried out by using a Micro‐infrared spectroscopy system (Bruker VERTEX70). Raman spectra were performed using ViaQontor (Renishaw) equipped with a 532 nm laser. A UV–vis near‐infrared spectrophotometer system (Lambd950) was used to measure optical absorption spectra. Current–voltage (*I–V*) curves were measured using a probe station equipped with a variable temperature platform and precision source/measure unit (Keysight, B2902A).

## Conflict of Interest

The authors declare no conflict of interest.

## Supporting information



Supporting Information

## Data Availability

The data that support the findings of this study are available from the corresponding author upon reasonable request.
