# Supplementary material for: Deep‐UV Light‐Emitting Based on the hBN:S/hBN: Mg Homojunction
Source: Adv Sci (Weinh). 2025 Mar 16;12(18):2414353. doi: 10.1002/advs.202414353 (PMC12079364; doi:10.1002/advs.202414353)
Supplement: Supplementary file 1 — Supporting Information [file ADVS-12-2414353-s001.docx]

Supporting Information

**Deep-ultraviolet light-emitting based on the hBN:S/hBN:Mg homojunction**

*Ransheng Chen, Qiang Li^*^, Wannian Fang, Qifan Zhang, Jiaxing Li, Zhihao Zhang, Kangkang Liu, Feng Yun, Yanan Guo^*^, Tao Wang^*^, Yue Hao**^*^*

Prof. Qiang Li

Key Laboratory of Physical Electronics and Devices for Ministry of Education and Shaanxi Provincial Key Laboratory of Photonics & Information Technology, Xi’an Jiaotong University, Xi’an, 710049, China

Email: [liqiang2014@mail.xjtu.edu.cn](mailto:liqiang2014@mail.xjtu.edu.cn)

Prof. Yanan Guo

State Key Laboratory of Solid-State Lighting, Institute of Semiconductors and Key Laboratory of Semiconductor Materials Science, Institute of Semiconductors,Chinese Academy of Sciences, Beijing, 100083, China

Email: [ynguo@semi.ac.cn](mailto:ynguo@semi.ac.cn)

Prof. Tao Wang

School of Physics and Astronomy, Cardiff University, Cardiff, CF24 3AA, UK

Email: [wangt61@cardiff.ac.uk](mailto:wangt61@cardiff.ac.uk)

Prof. Yue Hao

School of Microelectronics, Xidian University, Xi'an, 710126, China

Email: [yhao@xidian.edu.cn](mailto:yhao@xidian.edu.cn)


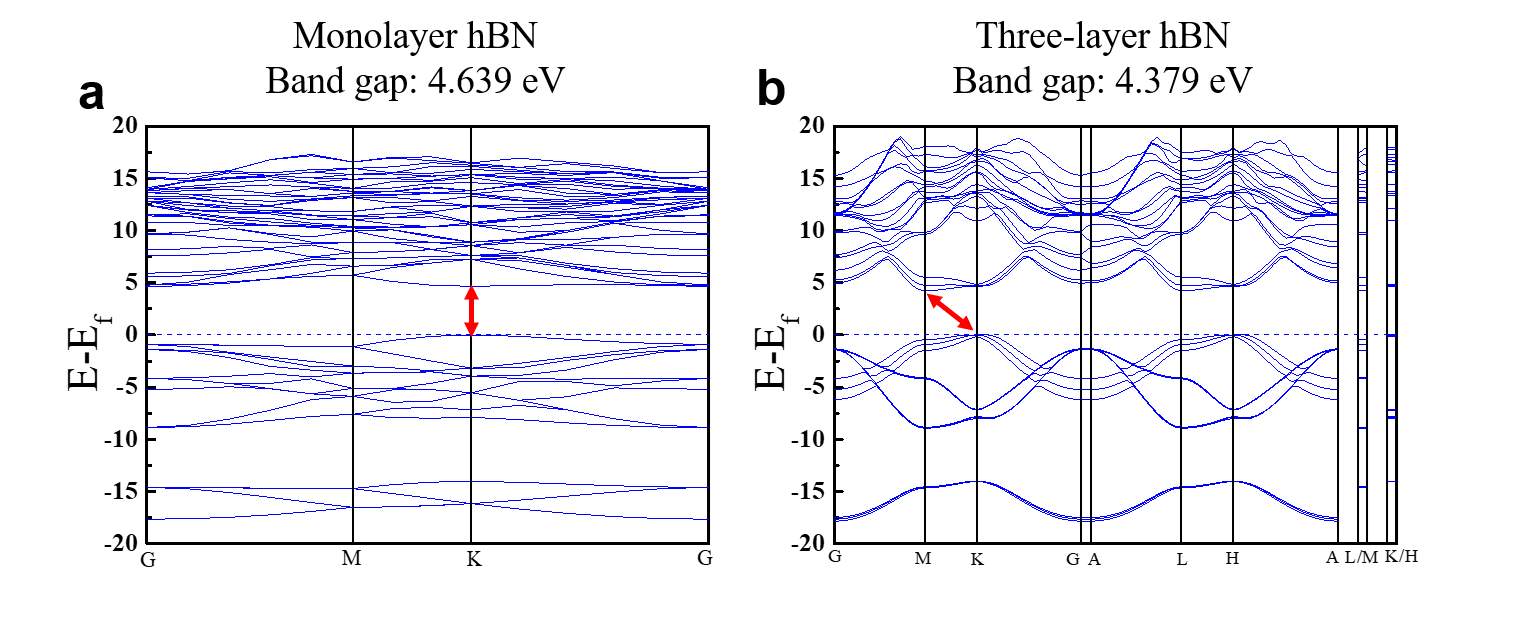


**Figure S1.** a) Electronic band structure of monolayer hBN film. b) Electronic band structure of three-layer hBN film.


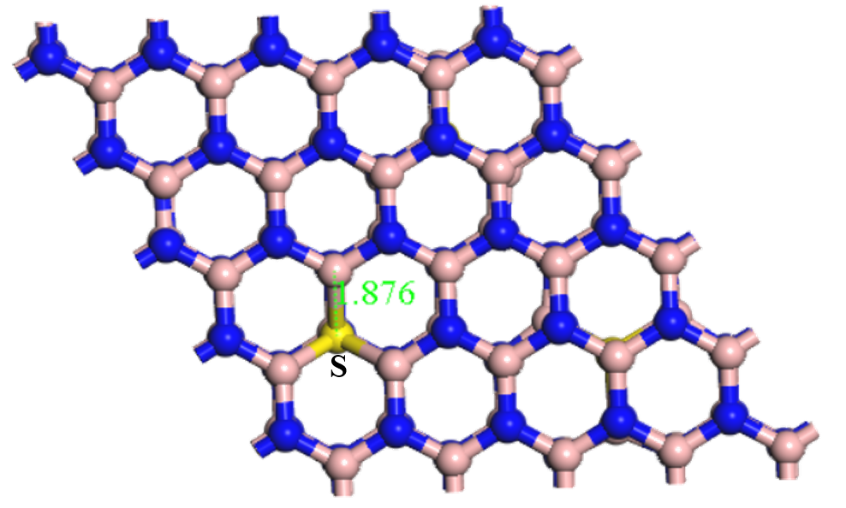


**Figure S2.** Simulated atomic structure of three layers S-doped hBN.


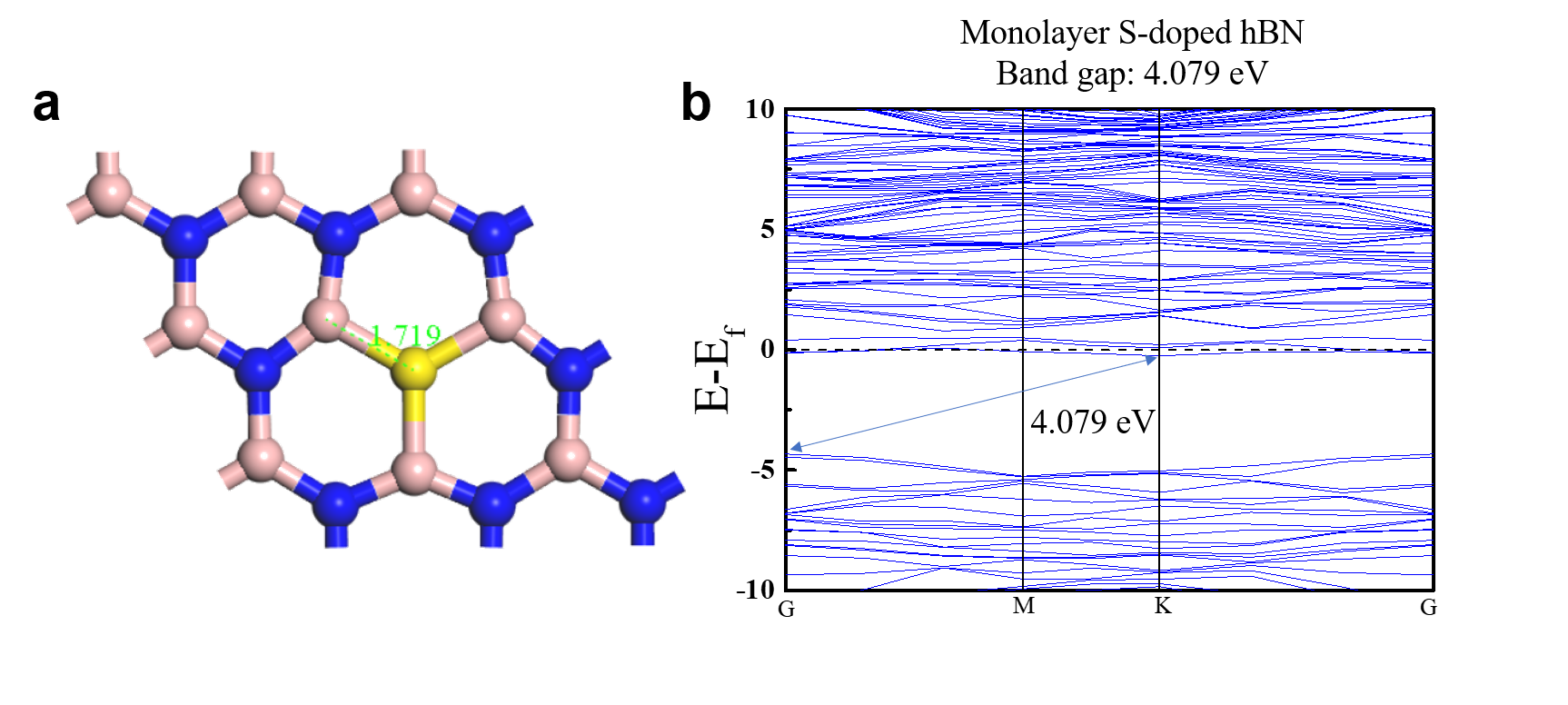


**Figure S3.** a) Simulated atomic structure of mono-layer S-doped hBN. b) Electronic band structure of mono-layer S-doped hBN.


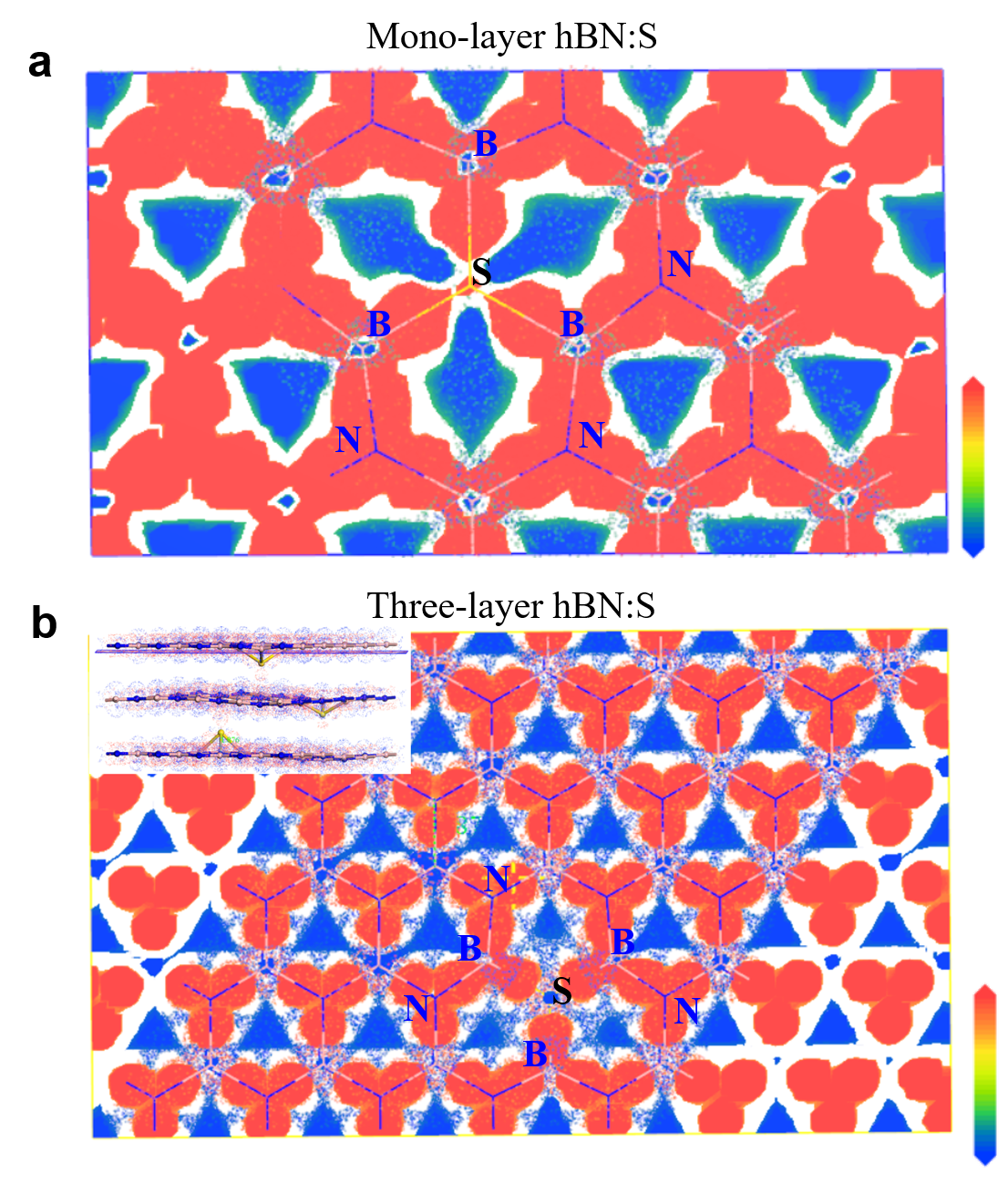


**Figure S4.** a) The 2D deformation charge density maps of mono-layer hBN:S. b) The 2D deformation charge density maps of three-layer hBN:S. The inset is the schematic diagram of the slice plane.


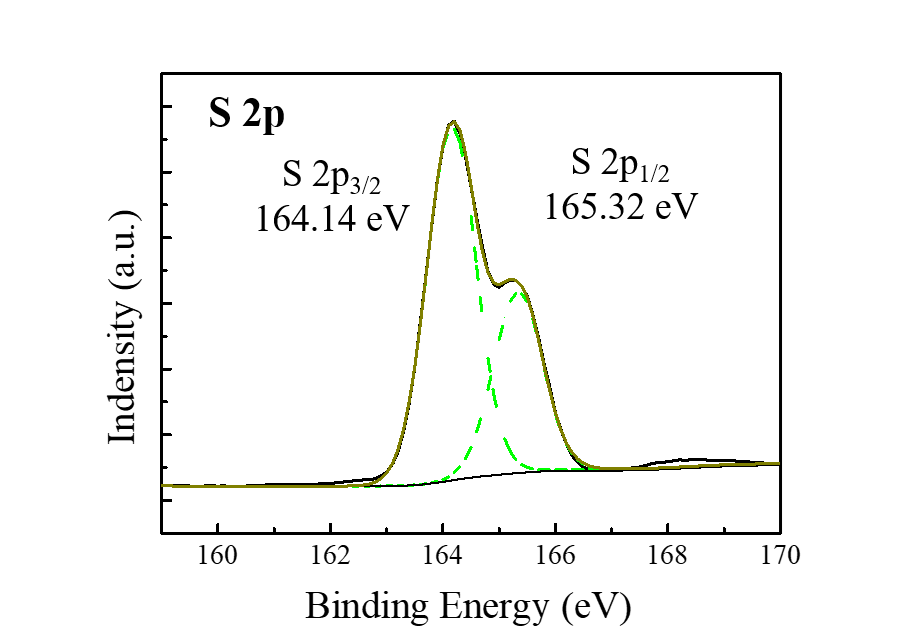


**Figure S5.** XPS spectra of S 2p core-level of S powder.


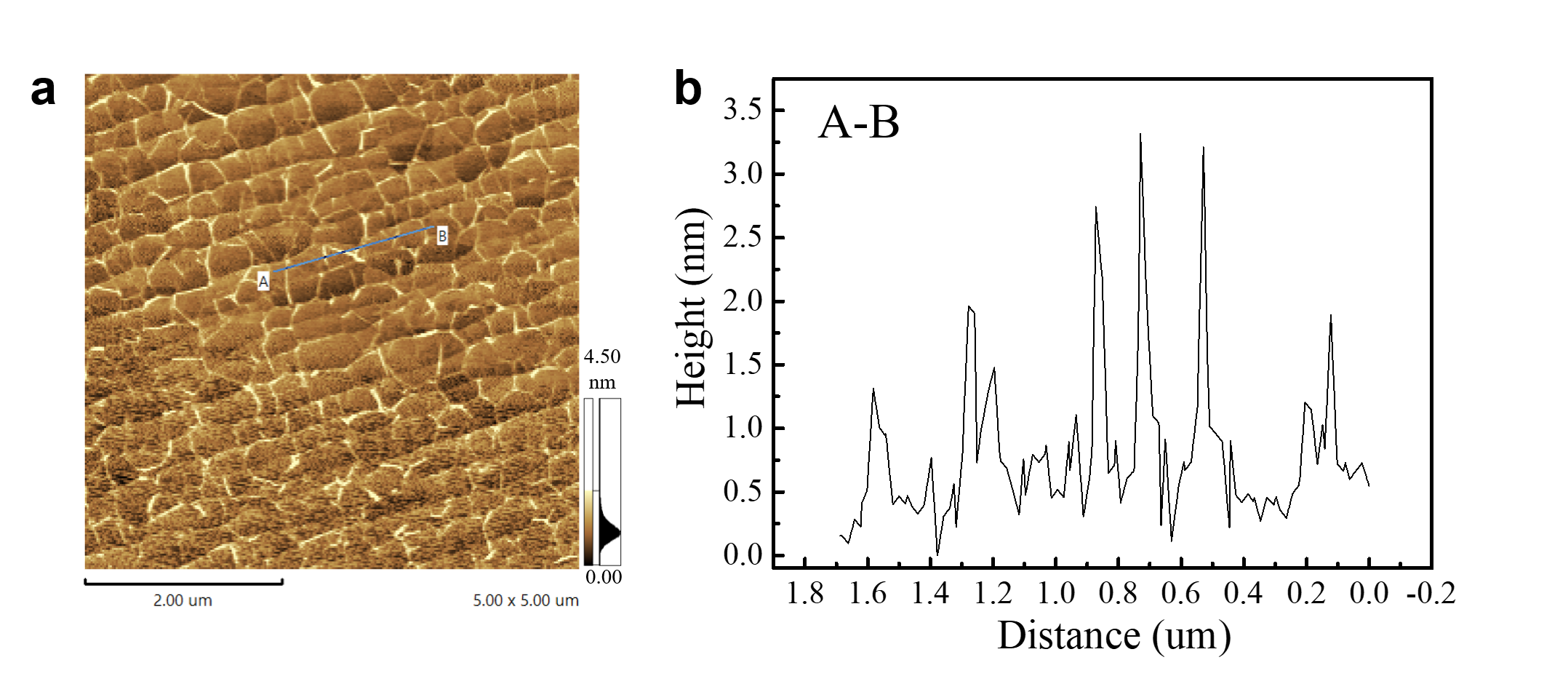


**Figure S6.** a) AFM image of hBN film on sapphire. b) The high profile along the A-B line marking.


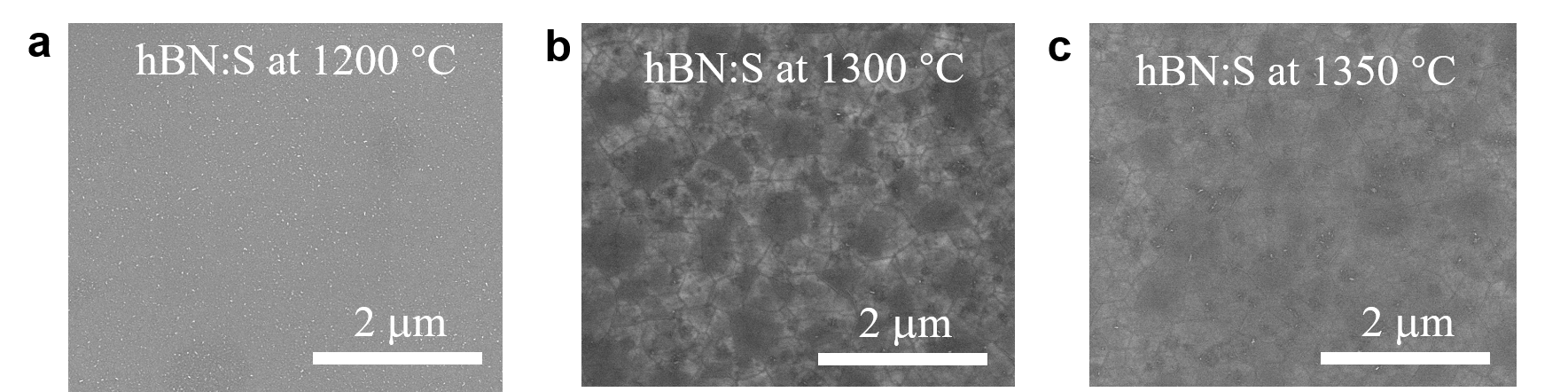


**Figure S7.** Top view SEM images of the hBN:S film synthesized with various growth temperatures: a) 1200 °C. b) 1300 °C. c) 1350 °C.


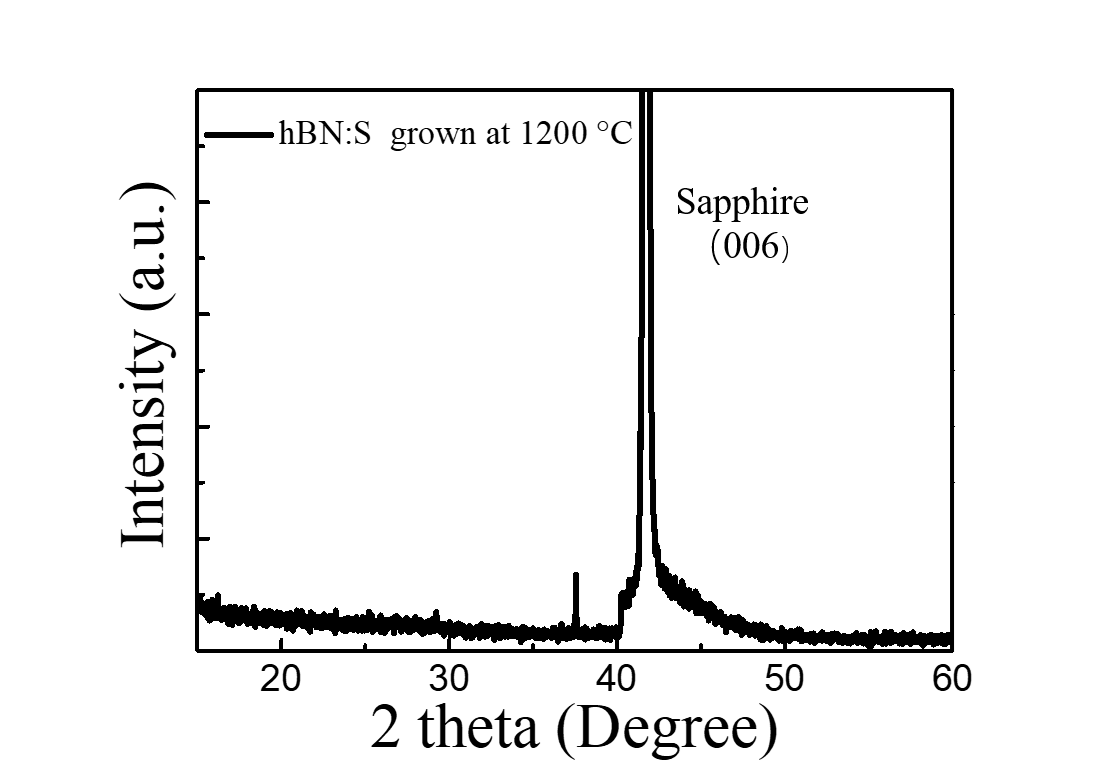


**Figure S8.** XRD characterization of hBN:S film synthesized at 1200 °C on a sapphire substrate.


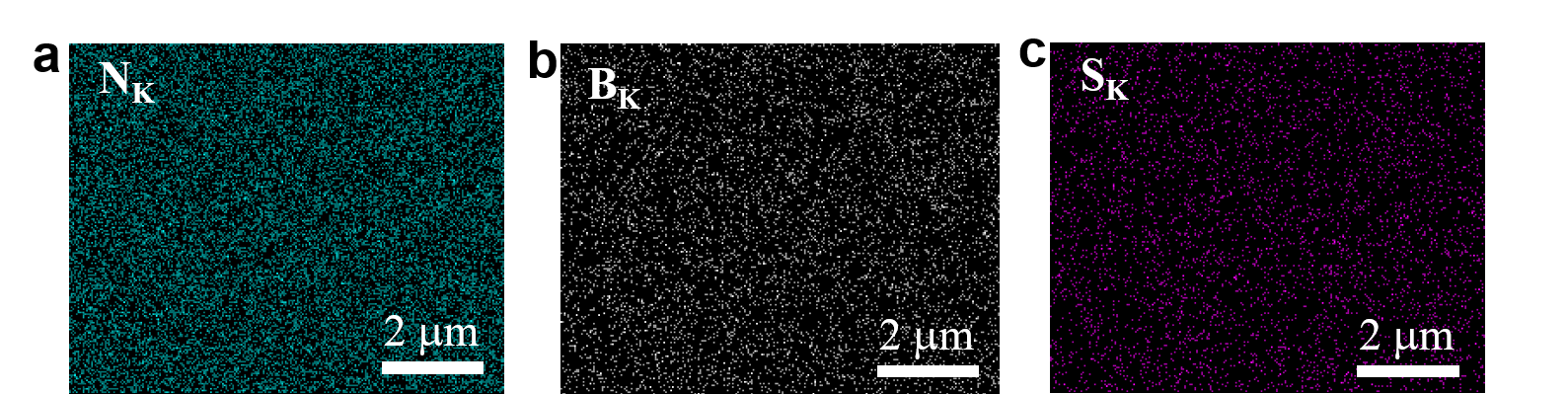


**Figure S9.** a-b) EDS mappings of a) N_K_, b) B_K_, c) S_K_ from hBN:S film grown at 1400 °C.


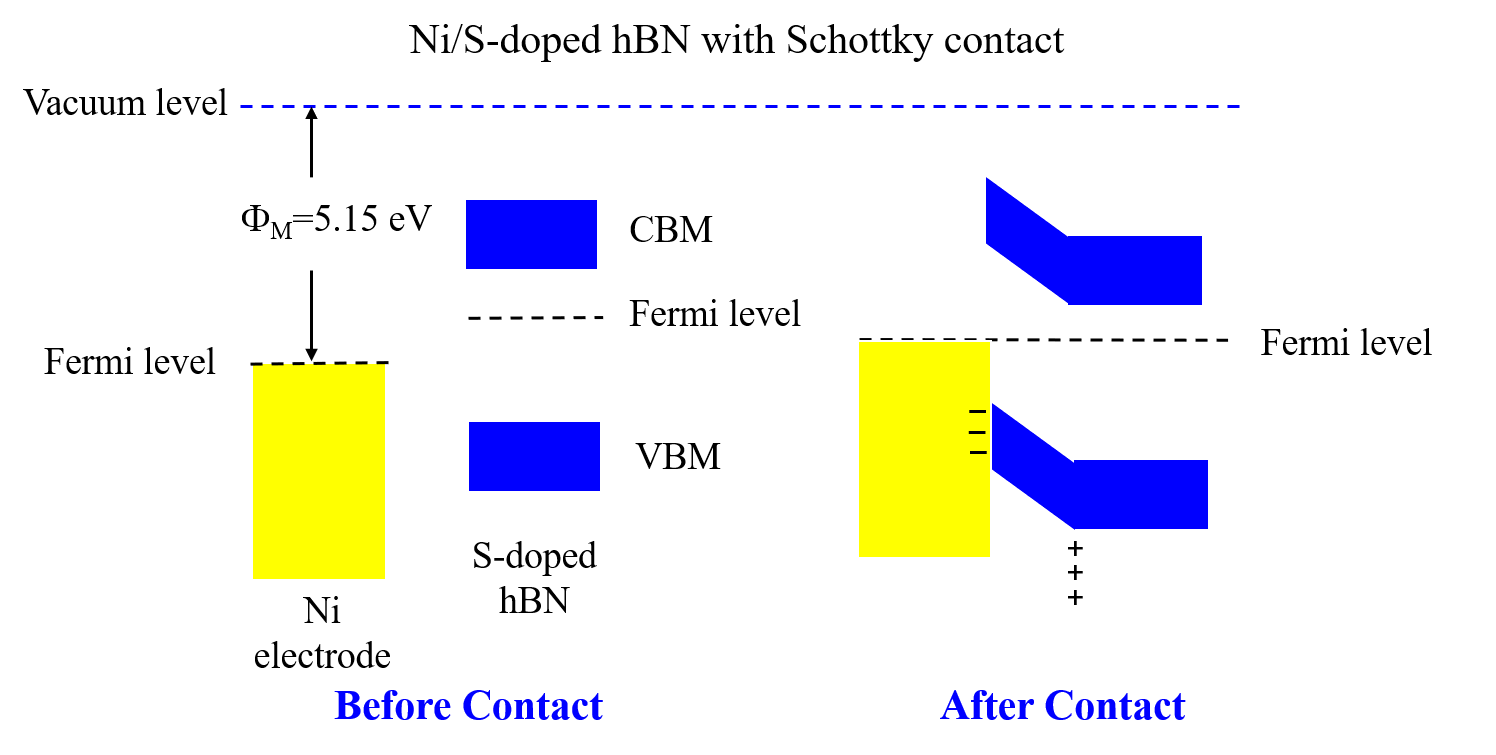


**Figure S10.** schematic layout of the Schottky contact between Ni and hBN:S film.


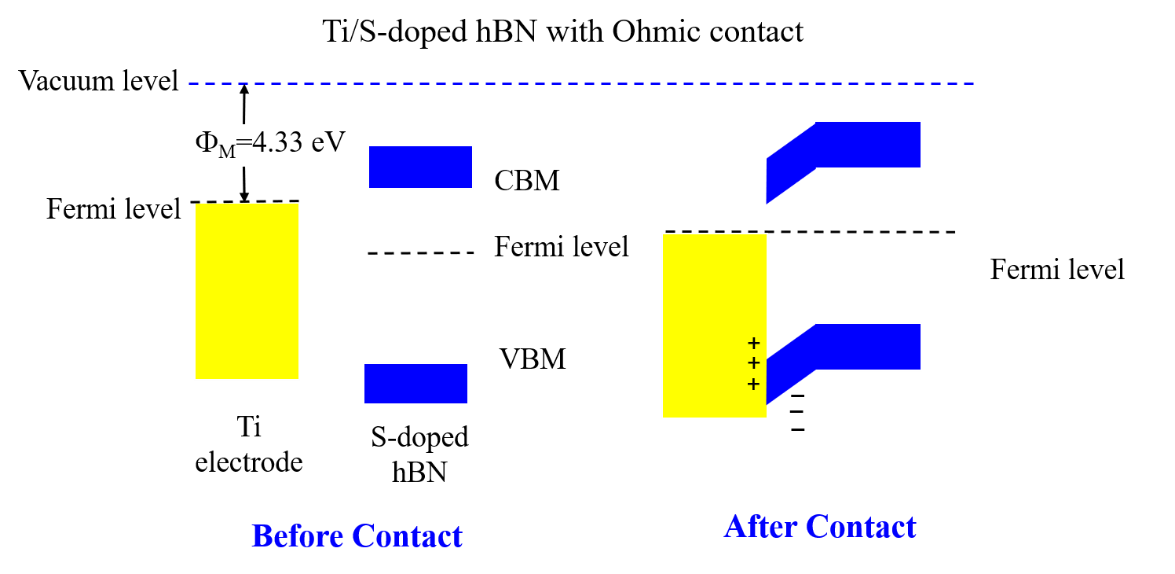


**Figure S11.** schematic layout of the Ohmic contact between Ti and hBN:S film.


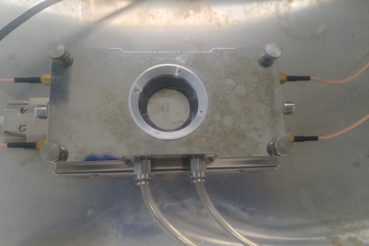


**Figure S12.** Variable-temperature I-V test platform.


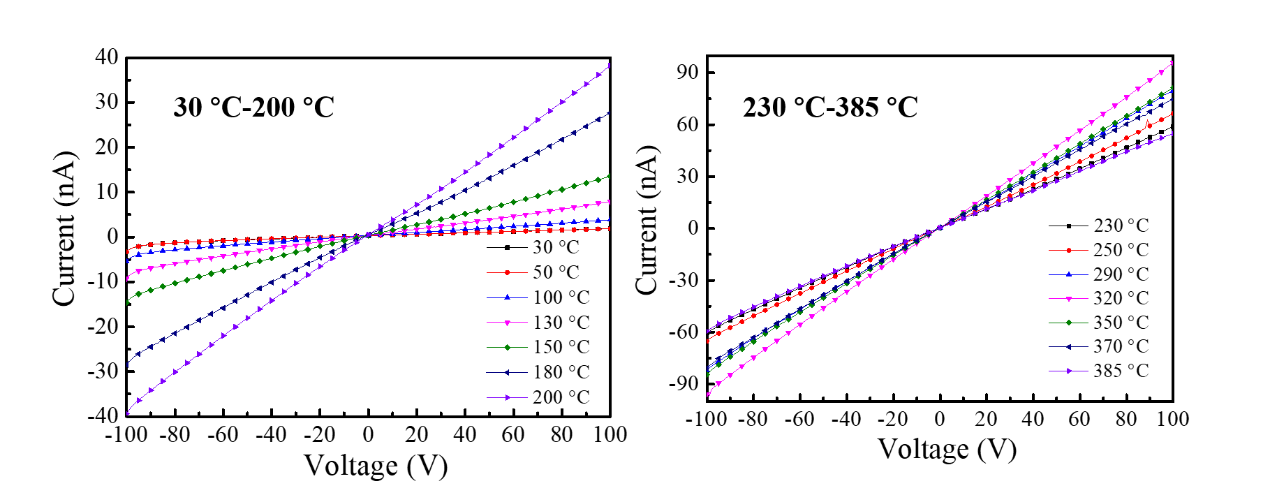


**Figure S13.** I-V characteristics of hBN:S samples on sapphire with Ti electrode, measured at different temperatures (30 °C-385 °C).

Under the low-temperature weak ionization region, conduction band electron concentration (n_0_) and the temperature satisfy the formula of $n_{0}\propto\exp(-\frac{\Delta E_{D}}{k_{0}T})$. Moreover, $R\propto\rho=\frac{1}{\sigma}=\frac{1}{n_{0}q\mu}\propto\exp(\frac{\Delta E_{D}}{k_{0}T})$, where the ρ and σ is resistivity and conductivity. So, we got the equation of$R=R_{0}\exp(\frac{\Delta E_{D}}{k_{0}T})$.


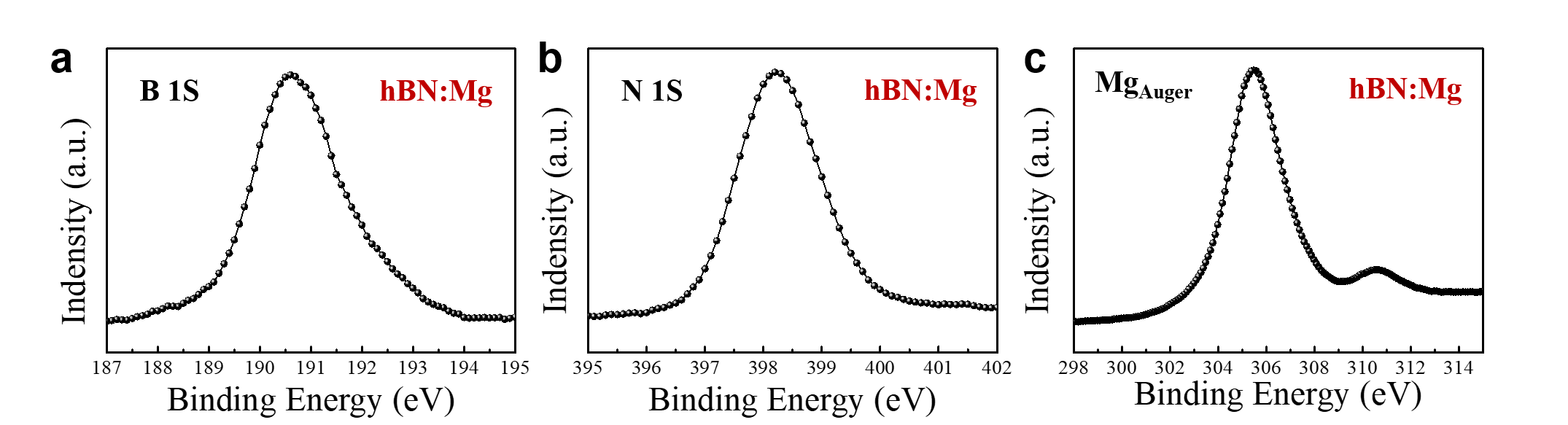


**Figure S14.** The XPS spectra were measured from a) B 1s, b) N 1s, and c) Mg_Auger_ core-level of hBN:Mg film.


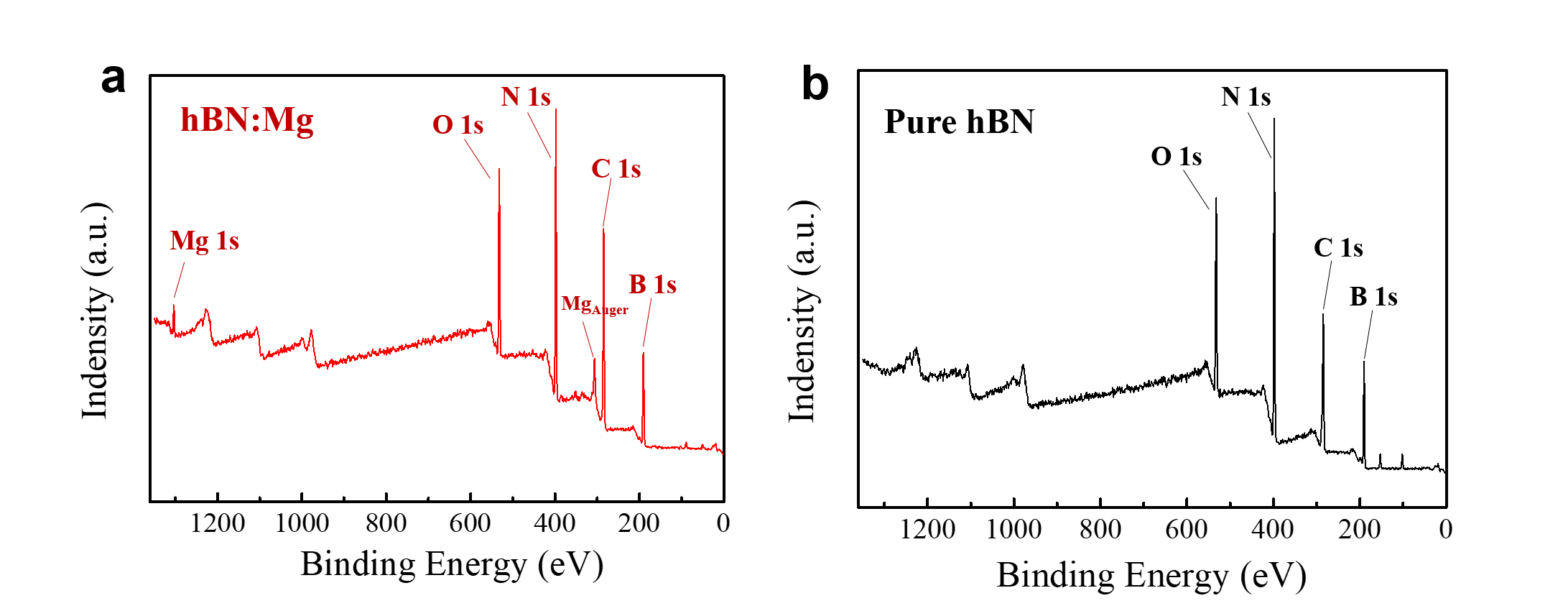


**Figure S15.** XPS survey spectra of a) Mg-doped hBN film, b) pure hBN film.


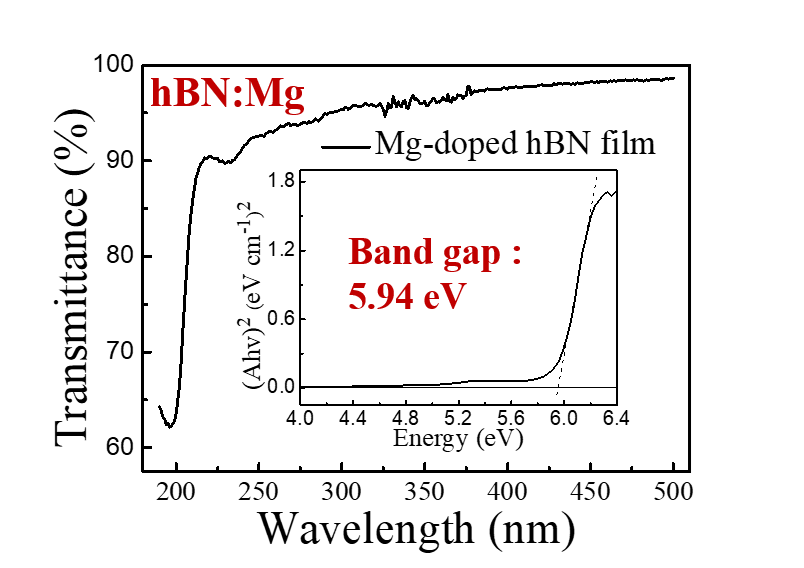


**Figure S16.** UV-vis absorption spectrum of hBN:Mg, which was transferred onto the sapphire. The inset is an optical band gap analysis of hBN:Mg films.


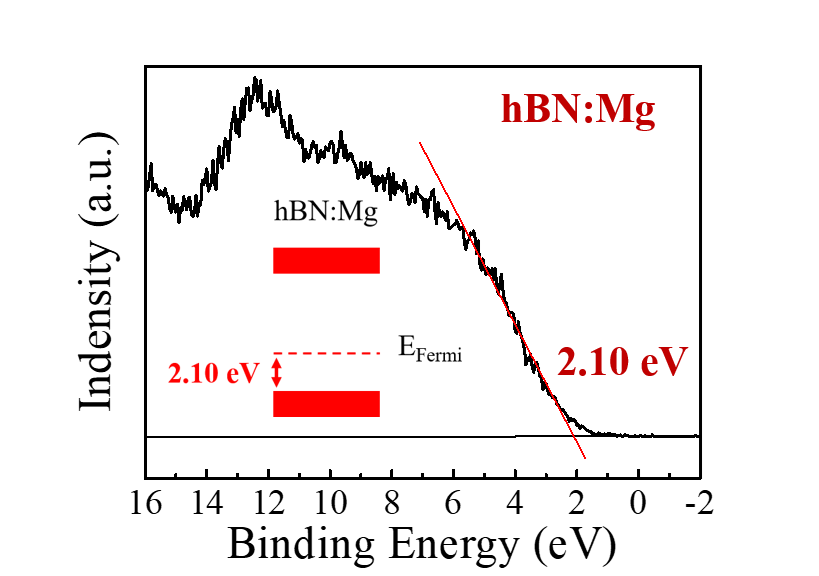


**Figure S17.** Valence band spectra of hBN:Mg film. The inset shows the schematic illustration of the band diagrams for hBN:Mg film.


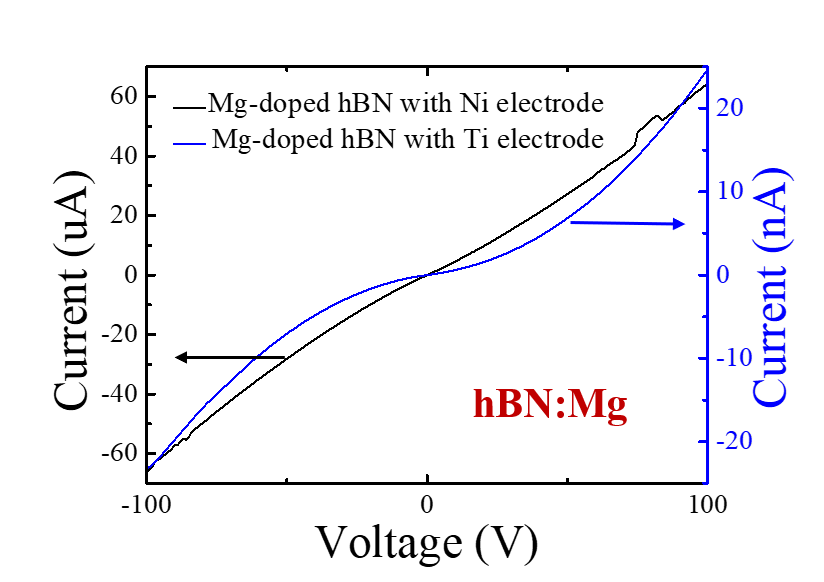


**Figure S18.** The I-V curve of the hBN:Mg film with Ni and Ti electrodes.


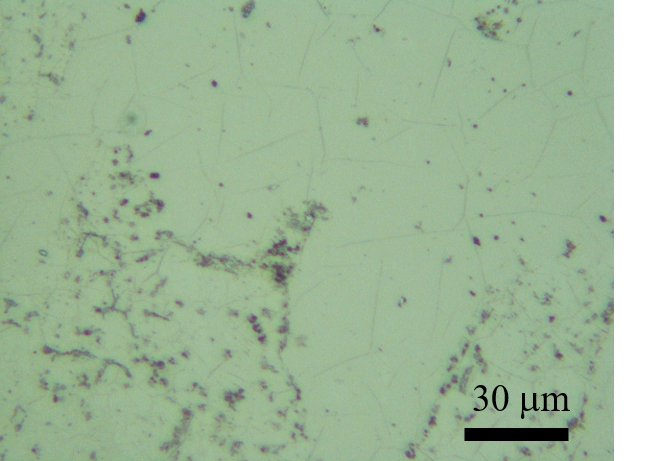


**Supplementary Figure 19.** The photograph of the surface morphology after the hBN:Mg film is transferred onto the hBN:S surface.


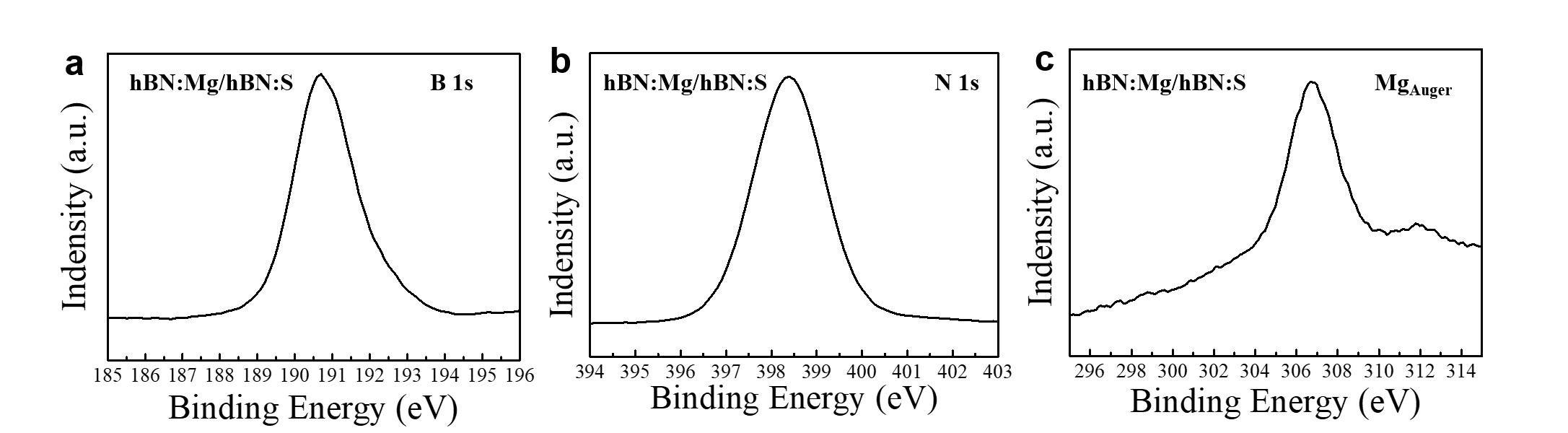


**Supplementary Figure 20.** The XPS spectra were measured from (a) B 1s, (b) N 1s, and (c) Mg_Auger_ core-level of hBN:Mg/hBN:S homojunction.


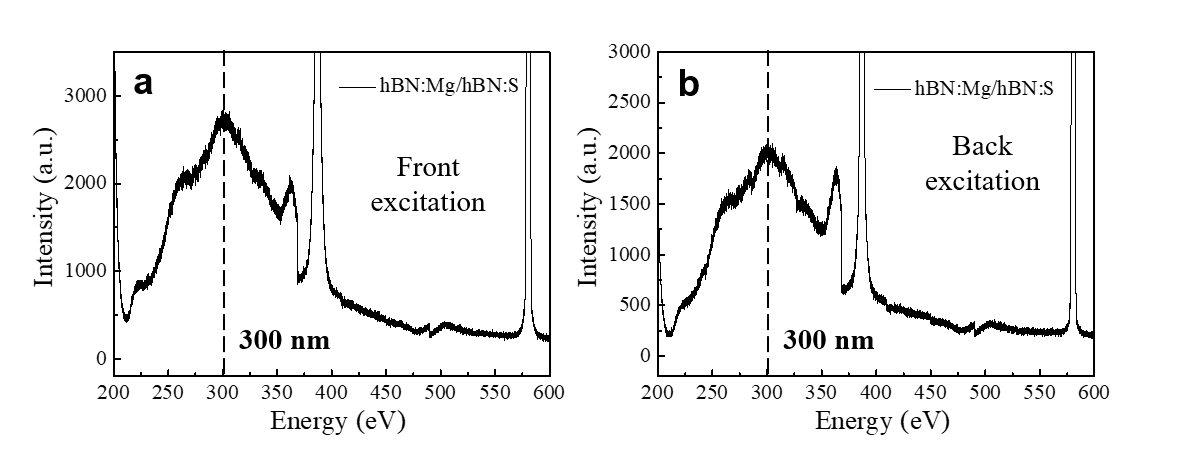


**Supplementary Figure 21.** The PL spectra of hBN:S/hBN:Mg homojunction from the front excitation (a) or the back excitation (b).


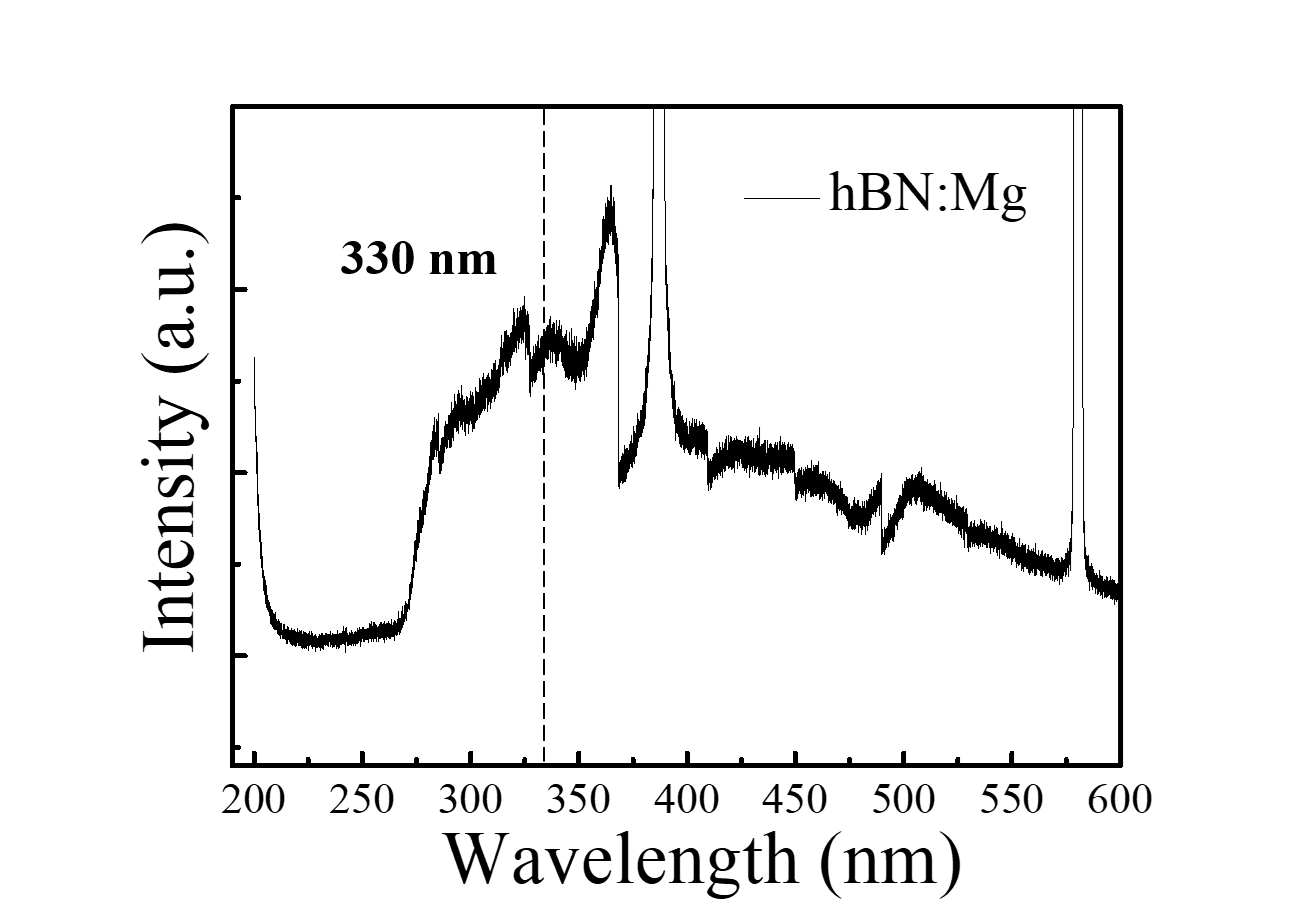


**Supplementary Figure 22.** The PL spectra of hBN:Mg film.
